# Demographic History and Inbreeding in Two Declining Sea Duck Species Inferred From Whole‐Genome Sequence Data

**DOI:** 10.1111/eva.70008

**Published:** 2024-09-10

**Authors:** María I. Cádiz, Aja Noersgaard Buur Tengstedt, Iben Hove Sørensen, Emma Skindbjerg Pedersen, Anthony David Fox, Michael M. Hansen

**Affiliations:** ^1^ Department of Biology Aarhus University Aarhus Denmark; ^2^ Danish Hunters' Association Rønde Denmark; ^3^ Department of Ecoscience Aarhus University Aarhus Denmark

**Keywords:** demographic history, effective population size, inbreeding, population decline, runs of homozygosity, whole‐genome sequencing

## Abstract

Anthropogenic impact has transitioned from threatening already rare species to causing significant declines in once numerous organisms. Long‐tailed duck (*Clangula hyemalis*) and velvet scoter (*Melanitta fusca*) were once important quarry sea duck species in NW Europe, but recent declines resulted in their reclassification as vulnerable on the IUCN Red List. We sequenced and assembled genomes for both species and resequenced 15 individuals of each. Using analyses based on site frequency spectra and sequential Markovian coalescence, we found *C*. *hyemalis* to show more historical demographic stability, whereas *M. fusca* was affected particularly by the Last (Weichselian) Glaciation. This likely reflects *C*. *hyemalis* breeding continuously across the Arctic, with cycles of glaciation primarily shifting breeding areas south or north without major population declines, whereas the more restricted southern range of *M. fusca* would lead to significant range contraction during glaciations. Both species showed evidence of declines over the past thousands of years, potentially reflecting anthropogenic pressures with the recent decline indicating an accelerated process. Analysis of runs of homozygosity (ROH) showed low but nontrivial inbreeding, with *F*_ROH_ from 0.012 to 0.063 in *C*. *hyemalis* and ranging from 0 to 0.047 in *M. fusca*. Lengths of ROH suggested that this was due to ongoing background inbreeding rather than recent declines. Overall, despite demographically important declines, this has not yet led to strong inbreeding and genetic erosion, and the most pressing conservation concern may be the risk of density‐dependent (Allee) effects. We recommend monitoring of inbreeding using ROH analysis as a cost‐efficient method to track future developments to support effective conservation of these species.

## 1 Introduction

Anthropogenic global change has led to dramatic declines or even extinctions in many species, fuelling the ongoing biodiversity crisis (Ceballos et al. 2015; Johnson et al. 2017). It is well established that rare species with restricted geographical distributions tend to have higher extinction risk as compared to more numerous and widespread species (Gaston 1998; Loiseau et al. 2020). However, even widespread species with large populations are no longer exempt from the risk of extinction, illustrated by the classic example of the passenger pigeon (*Ectopistes migratorius*), which was extirpated over a few decades as a result of unsustainable use and habitat destruction (Halliday 1980; Hung et al. 2014). Therefore, when widespread abundant species start to decline there can be cause for concern, even prior to reaching seemingly critical levels of decline. This is especially the case in animal populations subject to hunting, where it is important to identify critical parameters to support feedback monitoring to maintain their favourable conservation status. Recent developments in conservation genomics provide a wealth of methods that can be used to pursue these aims (Ellegren 2014; Ceballos et al. 2018; Allendorf et al. 2022).

Some of the most important developments concern the ability to analyse demographic history based on sequentially Markovian coalescent methods, site frequency spectra or combinations of these (Gutenkunst et al. 2009; Li and Durbin 2011; Schiffels and Durbin 2014; Terhorst, Kamm, and Song 2017; Beichman, Huerta‐Sanchez, and Lohmueller 2018; Liu and Fu 2020; Wang et al. 2020). Application of these methods shows that population size fluctuations are a regular feature of the long‐term demographic history of many species (Miller et al. 2012; Moura et al. 2014; Nadachowska‐Brzyska et al. 2015; Johnson et al. 2018). However, in some cases, evidence also suggests that current anthropogenic population declines are of unprecedented magnitude (Feng, Liu, and Hansen 2022). It is also now well established that inbreeding can have major effects on fitness and ultimately extinction risk (Saccheri et al. 1998; Keller and Waller 2002; Spielman, Brook, and Frankham 2004; O'Grady et al. 2006; Niskanen et al. 2020; Jackson et al. 2022). Hence, monitoring genetic parameters such as effective population size (*N*_e_) and heterozygosity can contribute important information about the conservation status of populations and species (Schwartz, Luikart, and Waples 2007; Andersson et al. 2022). Furthermore, our ability to estimate inbreeding coefficients using runs of homozygosity (ROH) analysis now allows for efficient monitoring of this crucial parameter (Ceballos et al. 2018; Kardos et al. 2018). In this context, it would be important to identify possible ‘tipping points’, where inbreeding significantly decreases fitness of populations. This will occur when masked genetic load, manifest as accumulated deleterious recessive alleles in heterozygotes, is converted into realised genetic load as a result of increased inbreeding, which will cause homozygosity for recessive deleterious alleles to decrease fitness (Dussex et al. 2023).

Many avian species have shown significant population declines in recent decades (Birdlife International 2022). Studies of endangered and extinct bird species (the latter using museum specimens) have yielded important new insights into their long‐term demographic history and shorter‐term inbreeding history (Hung et al. 2014; Nadachowska‐Brzyska et al. 2015; Murray et al. 2017; Gelabert et al. 2020; Duntsch et al. 2021; Mathur and DeWoody 2021; Robinson et al. 2021; Li et al. 2022; Femerling et al. 2023). For instance, the extinct Carolina parakeet (*Conuropsis carolinensis*) shows evidence of historical fluctuations in *N*_e_, likely reflecting glacial and interglacial periods. Surprisingly, however, ROH analysis does not provide evidence for strong inbreeding prior to extinction, suggesting drastic and sudden human‐mediated extirpation (Gelabert et al. 2020). The extant but critically endangered California condor (*Gymnogyps californianus*) also shows historical fluctuations of *N*_e_ with long‐term significant declines towards the present, but in contrast to the Carolina parakeet, numerous long ROHs are evident documenting strong recent inbreeding (Robinson et al. 2021).

Most studies to date have concerned endangered species showing relatively narrow distribution ranges. Here, we focus on two more widespread sea duck species, velvet scoter (*Melanitta fusca*) and long‐tailed duck (*Clangula hyemalis*), that nevertheless also show contrasts with respect to distributional ranges and census population sizes. Both species are now of conservation concern after having suffered population declines in recent generations (Hearn, Harrison, and Cranswick 2015; Dagys and Hearn 2018).

*Melanitta fusca* is a part of a complex of closely related species with similar ecology, behaviour and morphology that breed throughout the boreal forest and southern tundra biomes across the entire northern hemisphere, also comprised of the white‐winged scoter (*M. deglandi*) in North America and Stejneger's scoter (*M. stejnegeri*) in eastern Eurasia east of the Yenisey River (Kear 2005). All of these species overwinter in coastal regions, and *M. fusca* that winter in the Baltic Sea breed from western Siberia to northern Europe, around the Baltic Sea coasts, in the Scandinavian and Russian taiga and tundra biomes, with a tiny disjunct and presumably isolated population in Georgia. The latter implies some level of population structuring between different breeding habitats (Dagys and Hearn 2018; Paposhvili 2021). The total census population size has been roughly estimated at 141,000–268,000 mature individuals (https://www.iucnredlist.org/species/22724836/183801134). *Melanitta fusca* has experienced population declines of ca. 60% from 1988–1993 to 2007–2009, ascribed to a range of factors such as increased predation risk, reduced food availability, habitat degradation and high levels of entanglement in fishing nets. The decline has resulted in its current classification on the IUCN (International Union for the Conservation of Nature) Global Red List as VU (vulnerable) (Dagys and Hearn 2018; Birdlife International 2020).

*Clangula hyemalis* is an Arctic‐nesting species breeding continuously across the entirety of polar North America, Greenland and Eurasia and overwintering in near‐shore marine areas (Kear 2005). Individuals wintering in the Baltic Sea originate from western Siberia and northern Russia/Scandinavia, where extrapolated monitoring trends from 1993 to 2010 suggested an estimated decline equating to 59% over three generations (Hearn, Harrison, and Cranswick 2015). Entanglement in fishing nets, oil pollution and ship traffic disturbance are particularly responsible for increased mortality (Bellebaum et al. 2013; Larsson and Karlsson 2016). At its maximum, the total population size of *C. hyemalis* has been estimated at 3,700,000 individuals, of which ca. 1,600,000 currently occur in western Siberia and northern Russia/Scandinavia (data from https://wpe.wetlands.org). Despite its global abundance and stable populations in other parts of its range, *C. hyemalis* has been classified as VU on the IUCN Global Red List (Birdlife International 2018).

Both sea duck species studied here show a complex population structure, typical of many Anatidae species. *C. hyemalis* females are philopatric to both breeding and wintering areas, whereas males are considered philopatric with respect to wintering, but not breeding areas (Quillfeldt et al. 2022), resulting in the species being considered near panmictic across large geographical regions. This conclusion is supported by a study showing virtually no genetic differentiation at nuclear markers, but simultaneous differentiation in maternally inherited mitochondrial DNA in *C. hyemalis* (Wilson et al. 2016). Similar results of low nuclear genetic differentiation have been reported for three North American *Melanitta* species that are close relatives of *M. fusca* (Sonsthagen et al. 2019).

In the present study, we used *M. fusca* and *C. hyemalis* as representatives of relatively numerous, widespread species that nevertheless show evidence of strong current declines. We first produced draft genome assemblies for *M. fusca* and *C. hyemalis* and subsequently, whole‐genome resequenced 15 individuals from each species. Based on these data, we reconstructed demographic histories and analysed inbreeding based on ROH. Our first aim was to compare demographic histories of the two species. From a general perspective, we would expect more stability in *C. hyemalis* as compared to *M. fusca* due to the higher population size of *C. hyemalis* and its continuous breeding distribution across polar regions. Hence, cycles of glaciations and de‐glaciations may primarily have shifted breeding areas of *C. hyemalis* further south or north, respectively, without causing significant population declines, unless, for instance, abundance of their food items (mainly bivalves) also decreased due to lowered productivity during glacial periods. Conversely, the more fragmented and restricted distribution of *M. fusca* could have rendered it more susceptible to declines during glaciations.

Our second aim was to assess the current declines in the context of long‐term demographic histories; are the current declines occurring against a backdrop of long‐term declines, or have the species shown relative stability since the end of the last glaciation?

Our third aim was to estimate inbreeding. Have the recent declines led to detectable inbreeding, or are effective population sizes still sufficiently high to avoid this? Given their differences in census population sizes, we were particularly interested in contrasting the two species. We would not expect to find inbreeding in *C. hyemalis* with census population size in the millions and an assumed near‐panmictic population structure, but is inbreeding a concern for a species like *M. fusca* with considerably lower census population size? Finally, from a management perspective, we also aimed to establish a baseline of inbreeding values allowing for future monitoring of this crucial parameter.

## 2 Materials and Methods

### 2.1 Samples

A total of 16 individuals were sampled of each species in overwintering areas in Denmark, in each case encompassing 13 males and three females (see details in Table 1). The sampled individuals were obtained from Danish hunters between December 2017 and January 2019, shot during the open hunting seasons as part of a separate study run by the Danish Hunters' Association and Aarhus University on the distribution and feeding ecology of *C. hyemalis* and *M. fusca* (Petersen et al. 2019). Individuals were stored at −18°C and kept frozen until samples of muscle tissue were collected. Tissue samples were then stored in 96% ethanol at −18°C until DNA extraction.

**TABLE 1:** Overview of sampled individuals of long‐tailed duck (*Clangula hyemalis*) and velvet scoter (*Melanitta fusca*).

ID	Species	Location	Date sampled	Geographical coordinate	Sex	Age
DJ20180121_023A	*C. hyemalis*	Sejerø Bugt	21‐01‐18	55°51′ N 11°33′ E	F	JUV
DJ20180121_024A	*C. hyemalis*	Sejerø Bugt	21‐01‐18	55°51′ N 11°33′ E	M	AD
DJ20180121_025A	*C. hyemalis*	Sejerø Bugt	21‐01‐18	55°51′ N 11°33′ E	M	AD
DJ20180125_001A	*C. hyemalis*	South of Møn	25‐01‐18	54°54′ N 12°21′ E	M	AD
DJ20180125_002A	*C. hyemalis*	South of Møn	25‐01‐18	54°54′ N 12°21′ E	M	AD
DJ20180125_003A	*C. hyemalis*	South of Møn	25‐01‐18	54°54′ N 12°21′ E	F	AD
DJ20180125_004A	*C. hyemalis*	South of Møn	25‐01‐18	54°54′N 12°21′ E	M	AD
DJ20180125_005A	*C. hyemalis*	South of Møn	25‐01‐18	54°54′ N 12°21′ E	M	AD
DJ20180125_006A	*C. hyemalis*	South of Møn	25‐01‐18	54°54′ N 12°21′ E	M	AD
DJ20180125_007A	*C. hyemalis*	South of Møn	25‐01‐18	54°54′ N 12°21′ E	F	AD
DJ20180125_009A	*C. hyemalis*	South of Møn	25‐01‐18	54°54′ N 12°21′ E	M	AD
DJ20180125_010A	*C. hyemalis*	South of Møn	25‐01‐18	54°54′ N 12°21′ E	M	AD
DJ20180125_011A	*C. hyemalis*	South of Møn	25‐01‐18	54°54′ N 12°21′ E	M	AD
DJ20180125_012A	*C. hyemalis*	South of Møn	25‐01‐18	54°54′ N 12°21′ E	M	AD
DJ20180125_014A	*C. hyemalis*	South of Møn	25‐01‐18	54°54′N 12°21′ E	M	AD
DJ20180121_005A	*M. fusca*	Sejerø Bugt	21‐01‐18	55°51′ N 11°33′ E	M	AD
DJ20180121_006A	*M. fusca*	Sejerø Bugt	21‐01‐18	55°51′ N 11°33′ E	M	AD
DJ20180121_007A	*M. fusca*	Sejerø Bugt	21‐01‐18	55°51′ N 11°33′ E	M	AD
DJ20180121_008A	*M. fusca*	Sejerø Bugt	21‐01‐18	55°51′ N 11°33′ E	F	JUV
DJ20180121_009A	*M. fusca*	Sejerø Bugt	21‐01‐18	55°51′ N 11°33′ E	F	JUV
DJ20180121_010A	*M. fusca*	Sejerø Bugt	21‐01‐18	55°51′ N 11°33′ E	M	AD
DJ20180121_011A	*M. fusca*	Sejerø Bugt	21‐01‐18	55°51′ N 11°33′ E	M	AD
DJ20180121_012A	*M. fusca*	Sejerø Bugt	21‐01‐18	55°51′ N 11°33′ E	M	AD
DJ20180121_013A	*M. fusca*	Sejerø Bugt	21‐01‐18	55°51′ N 11°33′ E	M	AD
DJ20180121_014A	*M. fusca*	Sejerø Bugt	21‐01‐18	55°51′ N 11°33′ E	M	JUV
X_AU20171219_001A	*M. fusca*	Ebeltoft Vig	19‐12‐17	56°12′ N 10°37′ E	M	AD
X_AU20171219_002A	*M. fusca*	Ebeltoft Vig	19‐12‐17	56°12′ N 10°37′ E	M	AD
X_AU20171219_003A	*M. fusca*	Ebeltoft Vig	19‐12‐17	56°12′ N 10°37′ E	M	AD
X_AU20171219_004A	*M. fusca*	Ebeltoft Vig	19‐12‐17	56°12′ N 10°37′ E	M	AD
X_AU20171219_005A	*M. fusca*	Ebeltoft Vig	19‐12‐17	56°12′ N 10°37′ E	F	AD
VSD_REF	*M. fusca*	Aalborg Bugt	19‐01‐19	56°43′ N 10°41′ E	M	AD
LTD_REF	*C. hyemalis*	Fakse Bugt	20‐01‐19	55°10′ N 12°12′ E	M	AD

Abbreviations: AD, adults; F, females; JV, juveniles; M, males; REF, individuals used to sequence and assemble the reference genomes.

### 2.2 De Novo Assembly of Genomes

One *C. hyemalis* and *M. fusca* male were chosen for de novo sequencing and assembly, outsourced to Beijing Genomics Institute (BGI, Hong Kong). DNA was obtained by phenol–chloroform extraction. Sequencing was conducted using 10× Genomics de novo DNA library preparation (DNA fragmented to sizes between 100 and 200 kb) followed by 150 bp paired‐end sequencing on an Illumina HiSeq X‐Ten platform, aimed for a sequencing depth of approximately 80×. Assembly was conducted using the 10× Genomics Supernova v2.0 assembler software with default parameters.

### 2.3 Sex‐Linked Scaffolds

Birds have sex chromosomes, with females being the heterogametic sex (ZW system). As scaffolds from sex chromosomes could bias our analyses (Nursyifa et al. 2022), we aimed to identify and subsequently remove putative sex‐linked scaffolds. We explored the synteny of the draft genome assemblies of *C. hyemalis* and *M. fusca* compared to a mallard (*Anas platyrhynchos*) chromosome‐level reference genome assembly (GenBank Accession: GCF_015476345.1) using the minimap2 v2.24 software (Li 2018). Contigs were filtered using SAMtools v1.6 to remove unmapped, secondary and alignments with mapping quality <20. From the remaining alignments, we identified scaffolds mapped to sex chromosomes (Chromosome W, NC_051803.1; Chromosome Z, NC_051804.1), which were subsequently excluded from further analysis.

### 2.4 Whole‐Genome Resequencing

DNA was extracted from 15 individuals of each species using the E.Z.N.A. Tissue DNA Kit (OMEGA, Bio‐Tek, CA, USA) following the manufacturer's recommendations. Whole‐genome resequencing was outsourced to BGI and involved 150 bp paired‐end sequencing on the DNBseq platform, aimed at providing sequencing depths of 20×.

### 2.5 Mapping and SNP Calling

Raw reads were trimmed with Sickle v1.33 (Joshi and Sickle 2011) and then mapped to their corresponding reference genomes (GenBank accession: *C. hyemalis*: GCA_029619115.1 and *M. fusca*: GCA_029620185.1) using BWA mem v0.7.17 (Li and Durbin 2009). The software Picard Tools v2.26.3 (Broad Institute 2019) was then used to sort, mark and remove duplicated reads in the alignments prior to genotyping calls.

SNP calling in *C. hyemalis* and *M. fusca* was conducted using the *mpileup* and *call* functions implemented in BCFtools v1.13 (Danecek et al. 2021) to obtain sample‐specific SNPs with a minimum mapping quality of 30 and a minimum base quality of 25. Subsequently, the data set was filtered using VCFutils.pl and VCFtools v0.1.16 (Danecek et al. 2011) to remove sites with extremely high or low depth (thresholds determined visually from depth distribution of SNPs; Figure S1). This provided an initial filtering which was subsequently followed by individual or allele‐specific filtering as specified below. Indels and monomorphic sites were also removed. We subsequently changed individual genotypes with low read depth (<10) or low quality (<15) to missing and filtered to retain only biallelic SNPs with a QUAL score ≥30 and <10% missing genotypes. Finally, we discarded any SNPs not in Hardy–Weinberg equilibrium using the R script VCF_HWF (https://github.com/shenglin‐liu/VCF_HWF/blob/main/VCF_HWF.r). This script implements the test statistic $F_{\text{IS}}\sqrt{N}$ (Brown 1970), where $F_{\text{IS}}$ is Wright's fixation index within populations and N is the sample size. The statistic is distributed according to a standard normal distribution with mean of 0 and standard deviation of 1, and values > |1.96| are significant at the 5% level. Heterozygote excess leads to negative values and heterozygote deficit leads to positive values. Hence, the statistic tests deviations from Hardy–Weinberg equilibrium caused by deviating heterozygosity and was implemented in order to remove artefacts such as paralogs. The resulting species‐specific VCF files constituted the base data sets and were used as input for downstream analyses unless otherwise specifically mentioned. We subsequently filtered the base data sets for minor allele frequency (MAF) ≥0.05 to be used for principal component analysis (see below). We also performed additional filtering steps for the ROH analyses (see below).

Additionally, we performed SNP calling with the ‐v parameter in BCFtools *call* disabled to produce an ‘all sites’ data set containing both polymorphic and monomorphic sites. The data set was subsequently filtered to exclude indels, sites with extreme depth or low genotype quality and SNPs out of HWE as described previously. No filtering for missing data or MAF was performed.

### 2.6 Basic Statistics and Population Structure Analysis

We calculated two measures of genetic diversity from the ‘all sites’ data set. Nucleotide diversity (*π*) was calculated in windows of 10 kb for each species using PIXY 1.0 (Korunes and Samuk 2021) and subsequently, the raw counts were averaged across all windows to obtain a genome‐wide estimate. Genome‐wide observed heterozygosity was calculated as the number of heterozygous sites divided by the total number of genotyped sites for each individual. We evaluated possible genetic structure among the sampled individuals by performing principal component analyses (PCA) using the *prcomp* function implemented in R v3.5.1 (R Core Team 2021) based on the base data sets filtered for an MAF of 0.05.

### 2.7 Demographic Histories

We analysed demographic history using two methods based on the sequentially Markovian coalescent, PSMC (Li and Durbin 2011) and MSMC2 (Schiffels and Durbin 2014; Wang et al. 2020), and a third method based on site frequency spectra (SFS), Stairway Plot 2 (Liu and Fu 2020). PSMC and MSMC2 have their strengths in resolving long‐term demographic history but have limited power for resolving history during the past few thousand generations. In contrast, Stairway Plot 2 has better resolution of demographic history towards the recent past, but depending on overall genetic variation, it shows poorer resolution into the more distant past.

In PSMC analysis, demographic history is inferred on the basis of a consensus genome sequence of a single individual. A ‘psmcfa file’ was produced from each individual BAM file according to the authors' instructions (https://github.com/lh3/psmc). We used a minimum read depth filter of 7×, roughly equal to one‐third of the genome‐wide average, for both species and a maximum read depth of 45× for *C. hyemalis* and 40× for *M. fusca*, equalling twice the genome‐wide average for the respective species. Due to fragmented reference genomes, we only used scaffolds longer than 100 kb as recommended by Nikolic et al. (2020), corresponding to 84.02% of the genome of *M. fusca* and 71.48% of the genome of *C. hyemalis*. PSMC was run with default parameter settings, namely time intervals (‐p) were set to ‘4 + 25*2 + 4 + 6’ with maximum of 25 iterations (‐N). Although these settings were optimised for humans (Li and Durbin 2011), no adjustments were necessary to obtain the recommended >10 recombination events inferred under each time parameter, and splitting of the first time window (‐p ‘2 + 2 + 25*2 + 4 + 6’ and ‐p ‘1 + 1 + 1 + 1 + 25*2 + 4 + 6’) produced unrealistically high *N*_e_ estimates at the most recent time interval—a typical sign of model overfitting (Mather, Traves, and Ho 2020; Schiffels and Wang 2020). For each individual, a distribution of estimates was obtained by splitting the input file into chunks of 500,000 bp and randomly sampling with replacement 10 bootstrap data sets of equal size to the original data set, which was then used as input to PSMC.

MSMC2 analysis incorporates genome sequences from multiple individuals. For this purpose, we first generated single‐sample VCF and mask files from individual BAM files using the bamCaller.py script from MSMC Tools (https://github.com/jessicarick/msmc2_scripts). We included only scaffolds that exceeded N50 in each of the species (Table 2). Then, we phased the VCF files using read‐based phasing implemented in the software WhatsHap v.2.3 (Martin, Ebert, and Marschall 2023). In short, this type of phasing is independent of sample size and is conducted for each individual on the basis of reads using VCF and BAM files as input. While particularly developed for long read sequencing data, it is also useful for shorter reads as in this study. The specific procedures followed the authors' instructions (https://github.com/whatshap/whatshap). MSMC2 requires creating a mappability mask file for each reference genome, for which we used SNPable pipeline (http://lh3lh3.users.sourceforge.net/snpable.shtml). Finally, for each species, we input all of the individual‐phased VCF and mask files and the reference genome mappability mask to the generate_multihetsep.py script (MSMC Tools) to produce the final input files for MSMC analysis. MSMC2 was then run with default parameters.

For Stairway Plot 2, the analyses were based on the base data set for each species. The R script vcf2sfs.r (Liu et al. 2018) was used to construct folded SFS and Stairway Plot 2 was subsequently run using default parameters and with singletons included. For the parameter *L*, representing the total number of observed nucleic sites, we input the average number of genotyped sites per individual in the ‘all sites’ data set: 815.646.817 and 788.579.287 for *C. hyemalis* and *M. fusca*, respectively.

As there was a slight tendency towards genetic differentiation between males and females (see Section 3), only males (the majority of individuals in the samples) were used for MSMC2. However, since sample size is a critical factor for Stairway Plot 2, all 15 individuals were included.

For all analyses, we assumed a generation time of 6.01 years for *C. hyemalis* and 5.78 years for *M. fusca* (Bird et al. 2020). A wide range of mutation rates have been assumed in studies of birds (Nadachowska‐Brzyska et al. 2015; Gelabert et al. 2020; Robinson et al. 2021). We used a mutation rate estimated for collared flycatcher (*Ficedula albicollis*) (*μ* = 4.6 × 10^−9^ per generation) (Smeds, Qvarnstrom, and Ellegren 2016) but also plotted results using a commonly assumed mutation rate as estimated for humans (*μ* = 1.25 × 10^−8^ per generation) (Scally and Durbin 2012; Schiffels and Durbin 2014).

### 2.8 Runs of Homozygosity

Runs of homozygosity analyses were performed using the R package detectRUNS v.0.9.6 (Biscarini et al. 2019) with the MAF‐filtered base data set for each species. Prior to conducting the ROH analyses, we performed additional filtering of our SNP data to retain only the most accurate genotype calls. Hence, following the example of Balboa et al. (2024), we used GENMAP v.1.3.0 (Pockrandt et al. 2020) to estimate the mappability of each genome assembly by computing the uniqueness of 100 bp k‐mers while allowing for up to 2 mismatches and excluded all sites with a mappability score <1 from the analyses. Due to the fragmented nature of the two reference genomes, the analyses were conducted over the 100 longest scaffolds in each species, hence omitting shorter scaffolds that could induce bias in the lengths of detected ROH.

We detected ROH using the sliding windows approach with identical parameters for both species. The size of the scanning window, and likewise the minimum number of SNPs to constitute a ROH, was set to 100 SNPs with at least 1 SNP per 10,000 bp and a maximum of 100,000 bp between individual SNPs. To account for sequencing and genotyping errors, as well as missing data, which can break up longer ROH, we allowed for up to 8% of SNPs in a window to be heterozygous and up to 15% missing genotypes. The allowance of heterozygote genotypes in a window was determined by visualising ROH and inspecting the occurrence of gaps with varying threshold values while allowing for detection of short ROH >10,000 bp, as described in Tengstedt et al. (2024). To prevent overestimation of ROH lengths through faulty inclusion of heterozygous SNPs at the ends of an ROH, we enforced that a minimum of 5% of the overlapping windows covering an SNP should be homozygous for it to be included in an ROH, effectively trimming four SNPs off each end. To exclude short homozygous segments that may be present simply due to linkage, and to reduce the risk of detecting artefactual ROH from allowing a proportion of heterozygous genotypes in each window, the minimum length requirement of an ROH was set to 0.5 Mbp.

The detected ROH were categorised based on length; 0.5–1, 1–2 and 2–4 Mbp (no longer ROH were observed). Under the assumption of 1 Mbp = 1 cM, ROH within each length category are expected to have arisen due to inbreeding within the last 100–50 generations, 50–25 generations and 25–12.5 generations, respectively (Thompson 2013; Kardos et al. 2018). The inbreeding coefficient ($F_{\mathrm{ROH}}$) was calculated for each individual as: $F_{\mathrm{ROH}}=\frac{L_{\mathrm{ROH}}}{L_{\text{AUTO}}}$, where $L_{\mathrm{ROH}}$ is the total length of all ROH fulfilling the previously stated requirements and $L_{\text{AUTO}}$ refers to the total length of the autosome genome covered by SNPs. In the present case, the length of the genome corresponds to the total length of the 100 longest scaffolds: 230.1 Mbp for *C. hyemalis* and 437.5 Mbp for *M. fusca*. Even though ROH consequently were not analysed across the entire genomes, we nevertheless note that the results provide estimates of the genome‐wide distribution of ROH.

## 3 Results

### 3.1 Assembly

The genome assembly for *C. hyemalis* had an estimated genome size of 1.1 Gb, 36,831 scaffolds, raw coverage of 47.07× and a scaffold N50 value (i.e., scaffold length at which the sum of all scaffolds' lengths contain 50% of the total length) of 548.3 kb. The assembly of *M. fusca* had an estimated genome size of 1.2 Gb, 11,736 scaffolds, raw coverage of 58.88× and a scaffold N50 of 2 Mb. Genome assembly statistics for the two species are shown in Table 2. The sizes of both genomes are comparable to those of other bird species, such as mallard (1.2 Gb) (Li et al. 2021), domestic chicken (*Gallus gallus* 1.25 Gb), zebra finch (*Taeniopygia guttata* 1.25 Gb), American crow (*Corvus brachyrhynchos* 1.27 Gb), bald eagle (*Haliaeetus leucocephalus* 1.43 Gb), golden eagle (*Aquila chrysaetos* 1.48 Gb) and California condor (1.51 Gb) (Robinson et al. 2021).

**TABLE 2:** Summary of long‐tailed duck (*Clangula hyemalis*, accession number GCA_029619115.1) and velvet scoter (*Melanitta fusca*, accession number GCA_029620185.1) reference genome assemblies.

	Long‐tailed duck (*C. hyemalis*)	Velvet scoter (*M. fusca*)
Genome size (bp)	1,130,355,540	1,152,187,352
Number of scaffolds	36,831	11,736
Scaffold N50 (bp)	548,271	2,003,583
Number of contigs	81,141	33,295
Contig N50 (bp)	47,528	106,788
Max length (bp)	7,389,439	11,737,687
GC percentage	41	41.5

### 3.2 SNP Calling

Approximately 186 million raw reads (SD = 19 million reads) for *C. hyemalis* and ca. 160 million raw reads (SD = 37 million reads) for *M. fusca* per individual were generated for 15 individuals of each species. SNP calling yielded a total of 48,000,684 and 13,769,153 raw variants distributed on 36,593 and 11,358 scaffolds for *C. hyemalis* and *M. fusca*, respectively (see Table S1).

We found 2047 scaffolds for *C. hyemalis* and 1187 scaffolds for *M. fusca* that mapped to the sex chromosomes W (NC_051803.1) and Z (NC_051804.1) of mallard (detailed in Tables S2 and S3). After exclusion of these scaffolds and general quality control and filtering of SNPs (detailed in Table S1), the number of SNPs was reduced to 27,714,630 (*C. hyemalis*) and 4,033,801 (*M. fusca*). The data sets filtered for MAF of ≥0.05 yielded 13,088,309 (*C. hyemalis*) and 2,927,329 (*M. fusca*) SNPs. The data sets including only the 100 longest scaffolds for the ROH analyses encompassed 1,624,336 (*C. hyemalis*) and 1,287,414 (*M. fusca*) SNPs. Finally, the ‘all sites’ data set covered a total of 842,834,404 and 894,082,196 sites in *C. hyemalis* and *M. fusca*, respectively.

### 3.3 Genomic Diversity and Population Structure Analysis

The highest genetic diversity was found in *C. hyemalis* with a nucleotide diversity of 0.0061 and individual genome‐wide observed heterozygosity ranging from 0.0062 to 0.0064. In *M. fusca*, nucleotide diversity was estimated to 0.0026 and genome‐wide observed heterozygosity ranged between 0.0025 and 0.0028.

**FIGURE 1:**
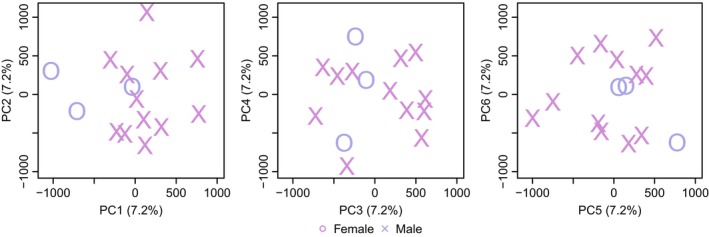
PCA for long‐tailed duck (*Clangula hyemalis*). Each point represents an individual with a shape denoting the sex (cross males; circle females).

**FIGURE 2:**
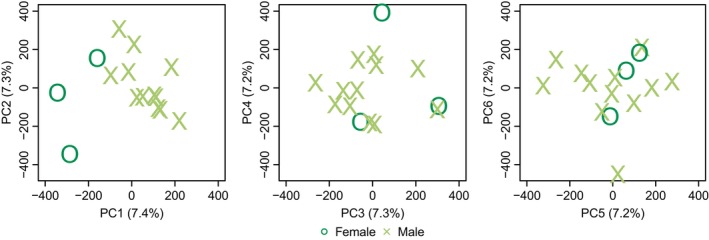
PCA for velvet scoter (*Melanitta fusca*). Each point represents an individual with a shape denoting the sex (cross males; circle females).

The PCA for *C. hyemalis* (Figure 1) and *M. fusca* (Figure 2) based on the MAF‐filtered base data set did not show obvious groupings of individuals, except for a slight tendency for separation between males (*N* = 12) and females (*N* = 3) along PC1, particularly in *M. fusca*. These small differences between males and females could potentially reflect remains from sex chromosome‐linked scaffolds or cryptic genetic structures reflecting sex‐biased dispersal.

### 3.4 Demographic Histories

**FIGURE 3:**
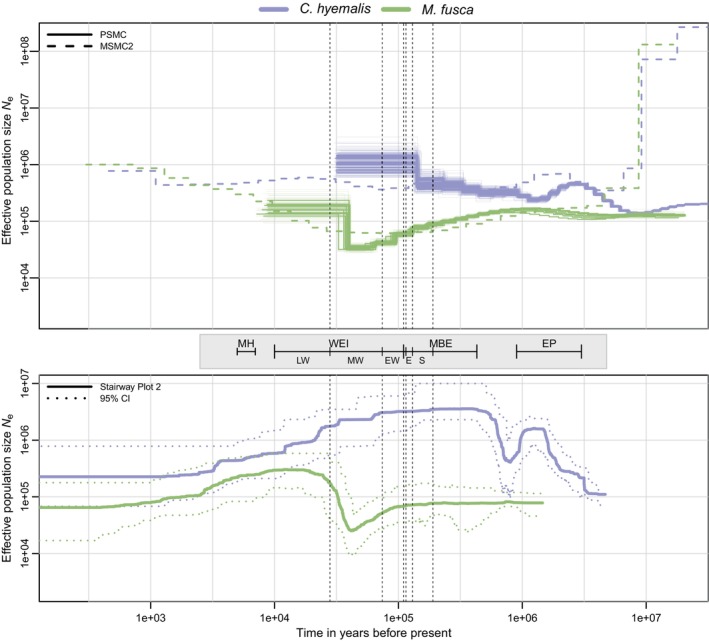
Reconstruction of the demographic history of long‐tailed duck (*Clangula hyemalis*, violet colour) and velvet scoter (*Melanitta fusca*, green colour). Top: Individual trajectories (thick lines) and bootstrapped trajectories (thin lines) for *C. hyemalis* and *M. fusca*, respectively, obtained by PSMC analysis, and population/species trajectories obtained by MSMC2 analysis. Bottom: Population/species trajectories with 95% confidence intervals based on Stairway Plot 2 analysis. All analyses used a mutation rate of 4.6 × 10^−9^ and a generation time of 6.01 years (*C. hyemalis*) and 5.75 years (*M. fusca*). The time intervals and dotted vertical lines denote important geological and climatic events: Early Pleistocene (EP, ca. 3–0.9 Myr); Mid‐Brunhes event (MBE, ca. 430–110 kyr); Saalian penultimate glaciation (S, ca. 190–130 kyr); Eemian interglacial (E, ca. 130–115 kyr); Weichselian glaciation (WEI, ca. 110–10 kyr); late Weichselian, (LW, ca. 28–10 kyr); middle Weichselian, (MW, ca. 74–28 kyr); early Weichselian (EW, ca. 110–74 kyr); and Mid‐Holocene (MH, ca. 7–5 kyr).

The demographic history trajectories of *C. hyemalis* and *M. fusca* inferred by PSMC, MSMC2 and Stairway Plot 2 and assuming *μ* of 4.6 × 10^−9^ are shown in Figure 3, whereas results assuming *μ* of 1.25 × 10^−8^ are shown in Figure S2. PSMC and MSMC2 showed congruent results, but with MSMC2 trajectories providing more information towards the present. Assuming *μ* of 4.6 × 10^−9^ (Figure 3), the results from *C. hyemalis* suggested ancient population expansions and declines, but relatively stable *N*_e_ for the past hundreds of thousands of years, even during the Weichselian (Last) Glaciation. *N*_e_ was around 5 × 10^5^ throughout the time period. *M. fusca* in contrast showed a gradual decline over the past hundreds of thousands of years, accelerating during the Weichselian Glaciation, followed by expansion towards the present. *N*_e_ was generally an order of magnitude lower than in *C. hyemalis*. MSMC2 showed increasing *N*_e_ in both species during the past few thousand years, but it should be noted that the method loses resolution towards the recent past. Assuming *μ* of 1.25 × 10^−8^ (Figure S2) suggested overall lower *N*_e_, in the order of a few hundreds of thousands in *C. hyemalis* and a few tens of thousands in *M. fusca*. The drastic decline in *M. fusca* also appeared later towards the end of the Weichselian Glaciation.

Long ROH could potentially bias PSMC‐like analyses. We did not observe ROH longer than 4 Mb in either species (see below), but nevertheless redid PSMC analyses with masked ROH for the three individuals in each species showing the highest *F*_ROH_. As shown in Figure S3, the results with and without masking of ROH were virtually indistinguishable.

The site frequency spectra (SFS) underlying the Stairway Plot 2 analyses showed L‐shapes in both species (Figure S4), albeit more distinct in *C. hyemalis* than in *M. fusca*, again indicating different demographic histories. This was also evident in Stairway Plot 2 analyses based on the SFS, which were largely congruent with PSMC and MSMC2 results, and furthermore, provided resolution into the more recent past. Assuming *μ* of 4.6 × 10^−9^ (Figure 3) *C. hyemalis* showed ancient population declines and expansions corresponding to those inferred by PSMC and MSMC2, albeit with considerably higher *N*_e_ during this time period inferred by Stairway Plot 2. A gradual decline occurred from ca. 1 × 10^5^ years ago stabilising at *N*_e_ of ca. 2 × 10^5^ towards the present. There was no clear imprint of events during the Weichselian Glaciation, although the gradual decline could be interpreted as a general effect of this glaciation. The demographic history of *M. fusca* was less resolved into the distant past compared to *C. hyemalis*, but showed strong decline to *N*_e_ of ca. 2.5 × 10^4^ occurring ca. 4 × 10^4^ years ago, followed by expansion to *N*_e_ of ca. 3 × 10^5^. Subsequently, for the past ca. 8 × 10^3^ years, *M. fusca* showed population decline to ca. 6.5 × 10^4^ at present. Assuming *μ* of 1.25 × 10^−8^ (Figure S2) dated the drastic historical decline in *M. fusca* to ca. 1.5 × 10^4^ years ago. The more recent decline was suggested to begin ca. 3 × 10^3^ years ago with *N*_e_ decreasing from ca. 1 × 10^5^ to ca. 2 × 10^4^. The recent decline in *C. hyemalis* started ca. 7 × 10^4^ years ago, with *N*_e_ decreasing from ca. 1.6 × 10^6^ to 9 × 10^4^ at present.

In total, the results from all three methods documented higher and more stable *N*_e_ in *C. hyemalis* than in *C. fusca*. Considering uncertainties of mutation rate and generation length, the most likely event underlying the historical decline in *M. fusca* is the Last Glacial Maximum ca. 2 × 10^4^ years ago. Stairway Plot 2, which provides better resolution of the recent past, suggested population declines in both species during the past thousands of years, most recently and prominently in *M. fusca*.

### 3.5 Runs of Homozygosity

**FIGURE 4:**
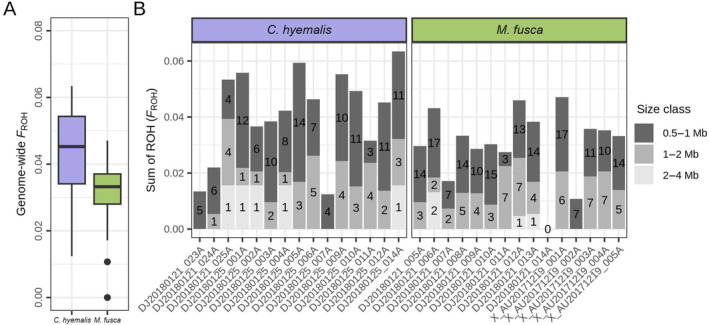
Genomic inbreeding levels in long‐tailed duck (*Clangula hyemalis*, violet) and velvet scoter (*Melanitta fusca*, green) as determined by runs of homozygosity (ROH) analyses. (A) Summary of individual inbreeding coefficients (*F*_ROH_) per species. (B) Individual size distribution of ROH and contribution to *F*_ROH_ from different ROH size classes. The *x*‐axis shows individuals in each species and the *y*‐axis shows the sum of ROH.

Even though the two species showed different demographic histories, the inferred patterns of inbreeding were quite similar. Between 8 and 20 ROH were detected per individual in *C. hyemalis* and between 0 and 26 in *M. fusca* (Table S4). This corresponded to inbreeding coefficients *F*_ROH_ of between 0.012 and 0.063 in *C. hyemalis* and 0 and 0.047 in *M. fusca* (Figure 4 and Table S5). Most detected runs were short, between 0.5 and 1 Mb and fewer between 1 and 2 Mb. Only four runs between 2 and 4 Mb were detected in *M. fusca* and five in *C. hyemalis* (Figure 4). In total, these results suggest low ongoing ‘background’ inbreeding rather than strong, recent inbreeding. It should be considered, however, that longer ROH could have been present but remained undetected or were fragmented due to the relatively short scaffold sizes.

This can be illustrated in plots of ROH for each scaffold and individual, where ROH were occasionally found at the beginning or end of scaffolds, suggesting they likely continue into adjoining scaffolds (Figures S5 and S6).

## 4 Discussion

Our population genomics analyses provide important new insights into the demographic histories and conservation status of the two species. *N*_e_ was consistently higher in *C. hyemalis* as compared to *M. fusca* and the latter was also more impacted by the Weichselian Glaciation. Both species showed indications of longer‐term population declines towards the present, and particularly for *M. fusca*, the recent strong declines could be seen as a continuation of these dynamics. Finally, despite differences in demographic histories, the two species showed similar patterns of inbreeding, both involving numerically low inbreeding coefficients. We discuss these findings and their biological and conservation implications below but first, highlight some technical and methodological aspects.

### 4.1 Technical Aspects and Challenges of the Analyses

Before discussing the biological implications of the results, we need to consider the reliability of results obtained by the different methods. The fragmented reference genomes could provide challenges for both the PSMC and MSMC2 analyses and for the analyses of inbreeding based on ROH. However, Patton et al. (2019) found PSMC and MSMC2 to be robust to fragmented reference genomes, and Nikolic et al. (2020) reached a similar conclusion for PSMC after removal of the shortest scaffolds. We consequently took steps to remove the shortest scaffolds for the PSMC and MSMC2 analyses. In the case of ROH, fragmented reference genomes could lead to runs being longer than the scaffolds, resulting in underestimation of ROH length. Also, short runs could be split between scaffolds and remain undetected, providing a downward bias of *F*_ROH_. In *M. fusca* and *C. hyemalis*, it was evident that inbreeding was low and runs consistently short, although we do not rule out the possibility that some runs were missed. Clearly, however, the fragmented reference genomes would have posed problems for more inbred species.

MSMC2 requires phasing, the accuracy of which depends on sample size (Browning and Browning 2011). We circumvented this problem by using read‐based phasing on an individual basis (Martin, Ebert, and Marschall 2023). However, it is worth noting that this method performs best with long‐read data, whereas our data consisted of 150 bp paired‐end reads. Despite this limitation, we assume that this has not severely affected results given the general congruence between PSMC and MSMC2 results. It should also be noted that putative population size changes obtained by these methods may instead reflect population structure (Mazet et al. 2016). Whereas this cannot be ruled out for the two sea duck species, the weak genetic structure observed in the species at present would suggest this to be a minor concern.

Sample sizes for Stairway Plot 2 were 15 in both species. Simulations by Liu and Fu (2015) using an earlier version of Stairway Plot showed that sample sizes of 30 chromosomes (corresponding to 15 individuals as in our study) showed lower accuracy and wider confidence intervals in recovering a complex population history, particularly towards the recent past, as compared to sample sizes of 90 and 180 chromosomes. Stairway Plot 2 would be expected to perform better than MSMC2 towards the recent past (Liu and Fu 2020). Therefore, while we emphasised the results from Stairway Plot 2, it is important to interpret these findings with caution given the relatively modest sample sizes.

### 4.2 Demographic Histories

The differences in demographic history trajectories and *N*_e_ estimates between the two species broadly agreed with our initial expectations. Hence, the widespread, numerous and arctic breeding *C. hyemalis* showed the highest *N*_e_ and nucleotide diversity, along with a limited response to climatic fluctuations during the Weichselian Glaciation. In contrast, the results suggested a stronger climatic impact on the more geographically restricted species *M. fusca*, largely restricted to boreal forest and tundra biomes. These results highlight the general point that species with superficially similar biology may show different patterns of genetic variability and demographic histories that can be linked to their ecology and geographical distribution. Similar differences in demographic histories between related species have been reported, for example, among three widespread grouse species: black grouse (*Tetrao tetrix*), willow grouse (*Lagopus lagopus*) and rock ptarmigan (*Lagopus muta*) (Kozma et al. 2016), as well as across species of penguins distributed from tropical to polar environments (Vianna et al. 2020).

The Stairway Plot 2 analyses suggested gradual declines in both species towards the present. In the case of *C. hyemalis*, this was suggested to have started ca. 1 × 10^5^ years ago and levelled out towards the recent past, thus likely not reflecting anthropogenic impact. The decline observed in *M. fusca* was more recent, starting within the last few thousand years. This raises the possibility that the decline at least partially reflects historical anthropogenic effects, and the current decline potentially represents an acceleration of ongoing longer‐term dynamics, as has been suggested in some other species (Hung et al. 2014; Nadachowska‐Brzyska et al. 2015; Wang et al. 2021).

### 4.3 Inbreeding

The two species showed similar patterns of inbreeding despite the differences in demographic histories and effective population sizes. *F*_ROH_ values were low (between 0% and 6% across individuals) but nevertheless nontrivial. This begs the question: Does the observed inbreeding reflect recent population declines? We argue that this is not the case, as ROH in both species were relatively short, indicating that they were not the result of strong recent inbreeding. How do we then interpret these inbreeding coefficients? Superficially, the assumption of mating structure of the species resulting in near panmixia over long geographical ranges combined with high effective population sizes, especially in *C. hyemalis*, would lead to expectations of *F*_ROH_ ≈ 0. However, the degree of population structure may have been underestimated, and there is a qualitative difference between strict panmixia as observed in only few widespread species (Als et al. 2011; Ulmo‐Diaz et al. 2023) and low but nevertheless biologically significant genetic differentiation that may be difficult to detect (Waples and Gaggiotti 2006). For the two sea duck species in the present study, genetic differentiation could occur if males after all show some degree of philopatry to reproductive sites, even if it is lower than for females; this would be expected to lead to higher inbreeding as compared to strict panmixia. Resolving this issue would require detailed analysis of individuals from different breeding areas rather than individuals sampled from overwintering sites as in the present study.

The advent of ROH analysis has significantly expanded possibilities for obtaining reliable estimates of inbreeding in wild populations (Ceballos et al. 2018; Allendorf et al. 2022). Understandably, there has been a tendency towards undertaking such studies for species of strong conservation concern. For example, ROH analyses of Scandinavian wolves (*Canis lupus*) and California condor have revealed particularly high *F*_ROH_ estimates of 0.54 and 0.26, respectively (Kardos et al. 2018; Robinson et al. 2021). Studies on presumably large and stable populations are less common, but Kardos, Qvarnstrom, and Ellegren (2017) provide estimates for *F*_ROH_ in populations of four flycatcher species: collared (*Ficedula albicollis*), pied (*F. hypoleuca*), Atlas (*F. speculigera*) and semicollared flycatchers (*F. semitorquata*). In all but one population, *F*_ROH_ was <0.03 but significantly different from zero, whereas in a recently founded population of collared flycatcher, *F*_ROH_ was ≈ 0.05. Based on these results, it does not seem unreasonable that *F*_ROH_ of similar magnitude observed in *C. hyemalis* and *M. fusca* represent low background inbreeding, especially if some degree of population subdivision is present.

Overall, despite the recent declines experienced by both species, there is no evidence to suggest that these declines have led to strong inbreeding and subsequent inbreeding depression at present. Conversely, however, the low inbreeding that nevertheless occurs would be expected to lead to some purifying selection against deleterious recessive alleles (Dussex et al. 2023), although it would be too low to lead to actual purging of deleterious alleles from the gene pools; both theory and empirical studies provide evidence that this would require much stronger inbreeding (Khan et al. 2021; Jackson et al. 2022; Dussex et al. 2023; Pecnerová et al. 2024).

### 4.4 Conservation Perspectives

Our results show that despite significant current declines, this has not led to strong inbreeding and genetic erosion at this point. Nevertheless, if declines persist, this is concerning as our results suggest that this could represent acceleration of longer‐term negative trends. Both species aggregate in larger numbers during certain life cycle stages and density‐dependent effects on breeding success have been observed (Hartman et al. 2013; Mineev et al. 2023). This points to Allee effects that come into play when population density decreases below a certain threshold to be a particular concern (Stephens, Sutherland, and Freckleton 1999; Hutchings 2015). Moreover, if declines continue, Allee effects could interact with genetic factors, such as emerging inbreeding depression or traits that represent adaptations for aggregating in large numbers but become maladaptive at lower population densities, further increasing risk of extinction (Murray et al. 2017).

From a general perspective, genetic monitoring, defined as analysing the same populations at different time points to estimate temporal genetic change, has proven a powerful design for following trends in populations and species of conservation concern (Schwartz, Luikart, and Waples 2007; Andersson et al. 2022). Monitoring so far has focused on genetic variation parameters such as allelic diversity, expected heterozygosity or in some cases, effective population size. However, rather than focusing on proxies, ROH analysis now enables monitoring the degree of inbreeding, a parameter of primary conservation concern. The results from *C. hyemalis* and *M. fusca* could be used to establish a baseline, and future sampling and sequencing efforts involving 10–15 individuals at regular time intervals could form the backbone of cost‐efficient and yet highly informative monitoring programmes in these and other species. Even though the two species have not yet declined to an extent where inbreeding is a concern, monitoring ROH could provide an important early warning about declines reaching thresholds where the viability of populations and species could be seriously compromised.

In total, the results of our study, along with other related studies (Kardos et al. 2018; Duntsch et al. 2021; Foote et al. 2021; Wang et al. 2021; Tengstedt et al. 2024), demonstrate how knowledge of the evolutionary and demographic history of species and populations obtained from genomic data can inform conservation. Current declines can be put into context by comparison to long‐term demographic trends and fluctuations. Furthermore, estimates of inbreeding obtained by ROH analysis provide crucial and direct information of conservation importance and allow for distinguishing between ongoing ‘background inbreeding’ within populations as opposed to recent inbreeding induced by population declines. In species showing complex migratory patterns and population dynamics like *C. hyemalis* and *M. fusca*, this provides valuable additional information that combined with ongoing population monitoring efforts can provide a more complete picture of their conservation status.

## Conflicts of Interest

The authors declare no conflicts of interest.

## Supporting information


Appendix S1.

## Data Availability

The draft genome assemblies of long‐tailed duck (*C. hyemalis*) and velvet scoter (*M. fusca*) are available from NCBI with the accession numbers GCA_029619115.1 and GCA_029620185.1, respectively. Raw reads for all analysed individuals have been deposited at NCBI with project number PRJNA796024. VCF files used for the different analyses have been uploaded to DRYAD (https://doi.org/10.5061/dryad.w3r22810z) (Cádiz et al. 2024). For each species, these encompass the raw SNP calls; the base data set containing SNPs filtered based on depth, quality, HWE and missing data; the base data set furthermore excluding singletons; and the base data set filtered to retain only SNPs located on the 100 longest scaffolds excluding singletons and low‐mappability sites. In addition, for each species, an ‘all‐sites’ file containing both monomorphic and polymorphic sites was filtered based on depth, quality and HWE.
