# Supplementary material for: Demographic History and Inbreeding in Two Declining Sea Duck Species Inferred From Whole‐Genome Sequence Data
Source: Evol Appl. 2024 Sep 10;17(9):e70008. doi: 10.1111/eva.70008 (PMC11386304; doi:10.1111/eva.70008)
Supplement: Supplementary file 1 — Appendix S1. [file EVA-17-e70008-s001.pdf]

Supplementary material

Demographic history and inbreeding in two declining sea duck species inferred from whole genome sequence data

María I. Cádiz, Aja Noersgaard Buur Tengstedt, Iben Hove Sørensen, Emma Skindbjerg Pedersen, Anthony David Fox & Michael M. Hansen

Supplementary tables

Table S1. Summary of SNP filtering in *C. hyemalis* and *M. fusca*. Bold text indicates the data sets that were used for downstream analyses.

	Clangula hyemalis		*Melanitta fusca*	
	**SNPs**	**Scaffolds**	**SNPs**	**Scaffolds**
Initial raw variant calls	48,000,684	36,593	13,769,153	11,358
No sites with extreme depth	38,833,229	33,501	11,081,095	9,147
Only bi-allelic SNPs	36,447,961	33,458	10,134,041	9,089
QUAL ≥ 30, minDP 10, minGQ 15	34,988,296	32,819	9,934,742	8,672
No sites with >10% missing data	29,911,580	30,110	4,467,828	5,498
No monomorphic sites	29,187,413	30,078	4,390,735	5,476
No sex chromosome scaffolds	28,336,623	28,550	4,173,165	5,065
**Only sites in HWE**	**27,714,630**	**28,363**	**4,033,801**	**4,849**
**MAF ≥ 0.05**	**13,088,309**	**27,519**	**2,927,329**	**4,530**
No sites with mappability <1	13,045,985	27,034	2,920,735	4,354
**Only 100 longest scaffolds**	**1,624,336**	**100**	**1,287,414**	**100**

Table S2. Summary synteny of *C. hyemalis* and *M. fusca* against *Anas platyrhynchos* (mallard).

	Clangula hyemalis	***Melanitta fusca***
Total (passed contigs + failed contigs)	196,995	170,820
Primary	36,831	11,736
Secondary	5,792	5,206
Supplementary	154,372	153,878
Mapped	188,191 (95.53 %)	166,687 (97.58%)
Primary mapped	28,027 (76.10 %)	7,603 (64.78%)
Filter	181,153	160,548

Table S3. Summary putative sexual scaffolds detected by the synteny of *C. hyemalis* and *M. fusca* against *Anas platyrhynchos* (mallard).

	Clangula hyemalis		***Melanitta fusca***	
	**Scaffolds**	**Length (bp)**	**Scaffolds**	**Length (bp)**
NC_051803.1 (Chromosome W)	460	18,239,449	425	29,756,918
NC_051804.1 (Chromosome Z)	1,691	89,514,817	877	94,346,700
NC_051803.1-NC_051804.1 (Chromosome WZ)	2047	99,671,612	1187	114,100,944

Table S4. Distribution of runs of homozygosity (ROH) by size in *C. hyemalis* and *M. fusca*.

	Clangula hyemalis		***Melanitta fusca***	
Size class (Mb)	**Number of ROH**	**Frequency**	**Number of ROH**	**Frequency**
0.5-1	122	75.8%	166	71.6%
1-2	34	21.1%	62	26.7%
2-4	5	3.1%	4	1.7%

Table S5. Individual inbreeding coefficients, F_{ROH}, in *C. hyemalis* and *M. fusca*.

**Individual**	**Species**	**Number of ROH**	**Sum of ROH**	F_{ROH}
DJ20180121_023A	*C. hyemalis*	5	3081081	0.0133
DJ20180121_024A	*C. hyemalis*	7	5066878	0.0219
DJ20180121_025A	*C. hyemalis*	9	12268184	0.0530
DJ20180125_001A	*C. hyemalis*	14	12839334	0.0555
DJ20180125_002A	*C. hyemalis*	8	8436522	0.0365
DJ20180125_003A	*C. hyemalis*	12	8850828	0.0383
DJ20180125_004A	*C. hyemalis*	10	9737382	0.0421
DJ20180125_005A	*C. hyemalis*	17	13653246	0.0590
DJ20180125_006A	*C. hyemalis*	12	10661734	0.0461
DJ20180125_007A	*C. hyemalis*	4	2853240	0.0123
DJ20180125_009A	*C. hyemalis*	14	12727878	0.0550
DJ20180125_010A	*C. hyemalis*	14	11347852	0.0490
DJ20180125_011A	*C. hyemalis*	7	7259269	0.0314
DJ20180125_012A	*C. hyemalis*	13	10414714	0.0450
DJ20180125_014A	*C. hyemalis*	15	14594267	0.0631
DJ20180121_005A	*M. fusca*	17	12979240	0.0297
DJ20180121_006A	*M. fusca*	21	18890484	0.0432
DJ20180121_007A	*M. fusca*	9	7501028	0.0171
DJ20180121_008A	*M. fusca*	19	14593954	0.0334
DJ20180121_009A	*M. fusca*	14	12524852	0.0286
DJ20180121_010A	*M. fusca*	18	13259654	0.0303
DJ20180121_011A	*M. fusca*	10	11996989	0.0274
DJ20180121_012A	*M. fusca*	21	20083174	0.0459
DJ20180121_013A	*M. fusca*	19	16783771	0.0384
DJ20180121_014A	*M. fusca*	0	0	0
X_AU20171219_001A	*M. fusca*	23	20589914	0.0471
X_AU20171219_002A	*M. fusca*	7	4683885	0.0107
X_AU20171219_003A	*M. fusca*	18	15633080	0.0357
X_AU20171219_004A	*M. fusca*	17	15440957	0.0353
X_AU20171219_005A	*M. fusca*	19	14538253	0.0332

Supplementary figures

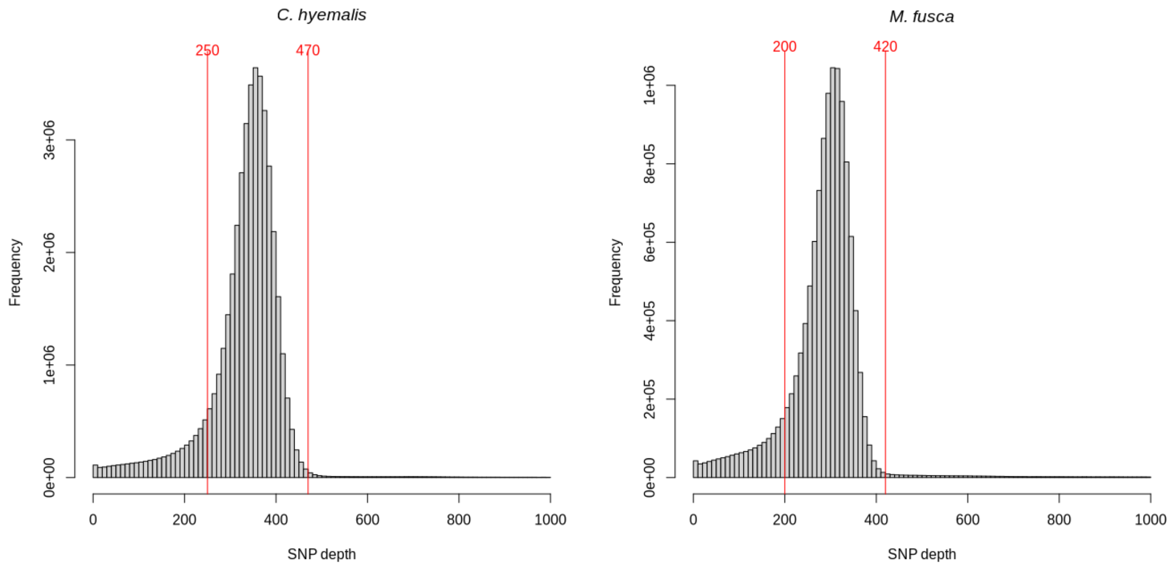

Fig. S1. Distribution of depth of coverage per SNP across all individuals in *C. hyemalis* and *M. fusca*, respectively. Vertical lines in red indicate the thresholds applied in the initial filtering process to remove sites with extremely high or low depth.

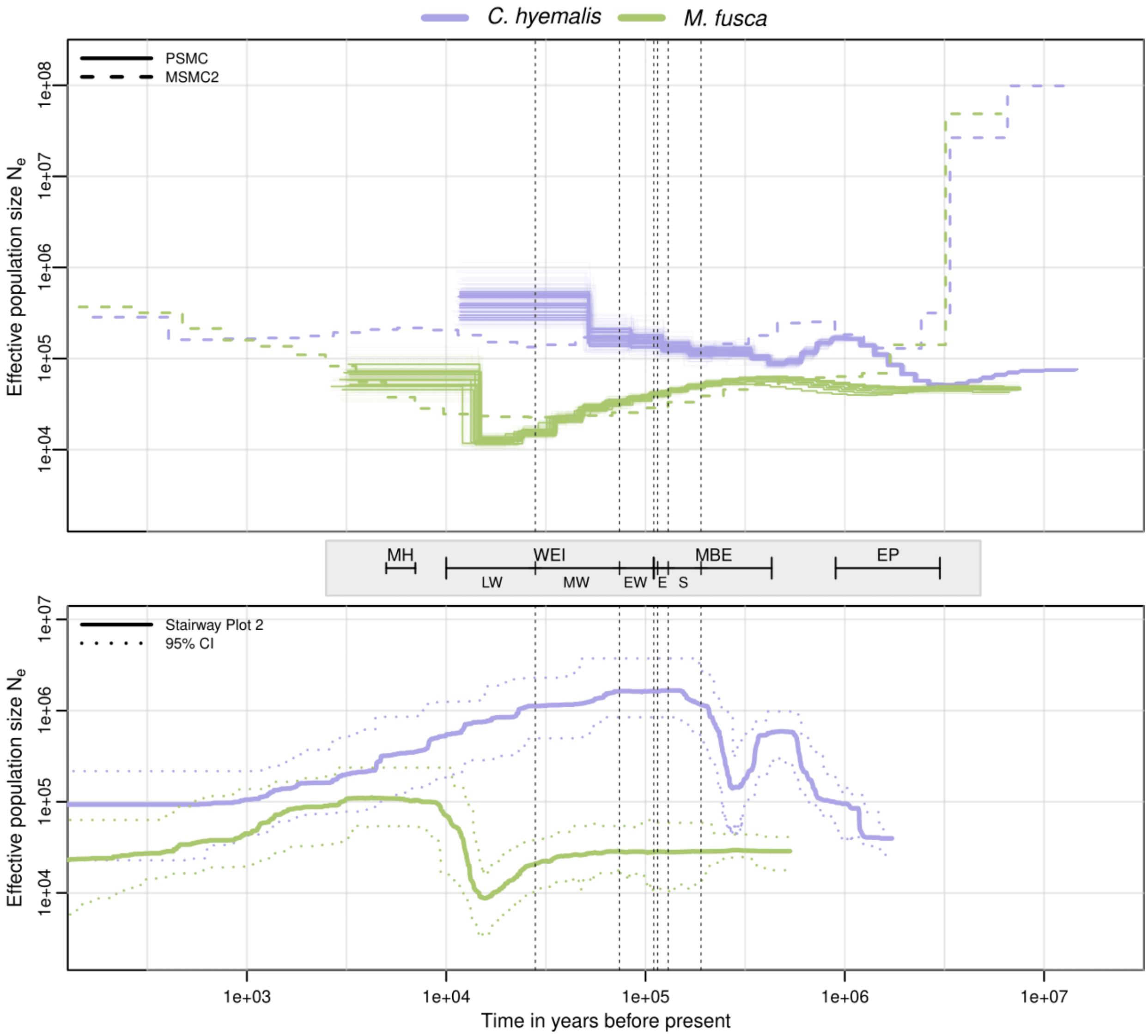

Figure S2. Reconstruction the demographic history of long-tailed duck (*C. hyemalis*, violet color) and velvet scoter (*M. fusca*, green color). Top: Individual trajectories (thick lines) and bootstrapped trajectories (thin lines) for *C. hyemalis* and *M. fusca*, respectively, obtained by PSMC analysis, and population/species trajectories obtained by MSMC2 analysis. Bottom: Population/species trajectories with 95% confidence intervals based on Stairway Plot2 analysis. All analyses used a mutation rate of 1.25 x 10^{-8} and a generation time of 6.01 years (*C. hyemalis*) and 5.75 (*M. fusca*). The time intervals and dotted vertical lines denote important geological and climatic events: Early Pleistocene (EP, ca. 3 - 0.9 Myr); Mid-Brunhes event (MBE, ca. 430 – 110 kyr); Saalian penultimate glaciation (S, ca. 190 – 130 kyr); Eemian interglacial (E, ca. 130 – 115 kyr); Weichselian glaciation (WEI, ca. 110 – 10 kyr); late Weichselian, (LW, ca. 28 – 10 kyr); middle Weichselian, (MW, ca. 74 – 28 kyr); early Weichselian (EW, ca. 110 – 74 kyr); Mid-Holocene (MH, ca. 7 – 5 kyr).

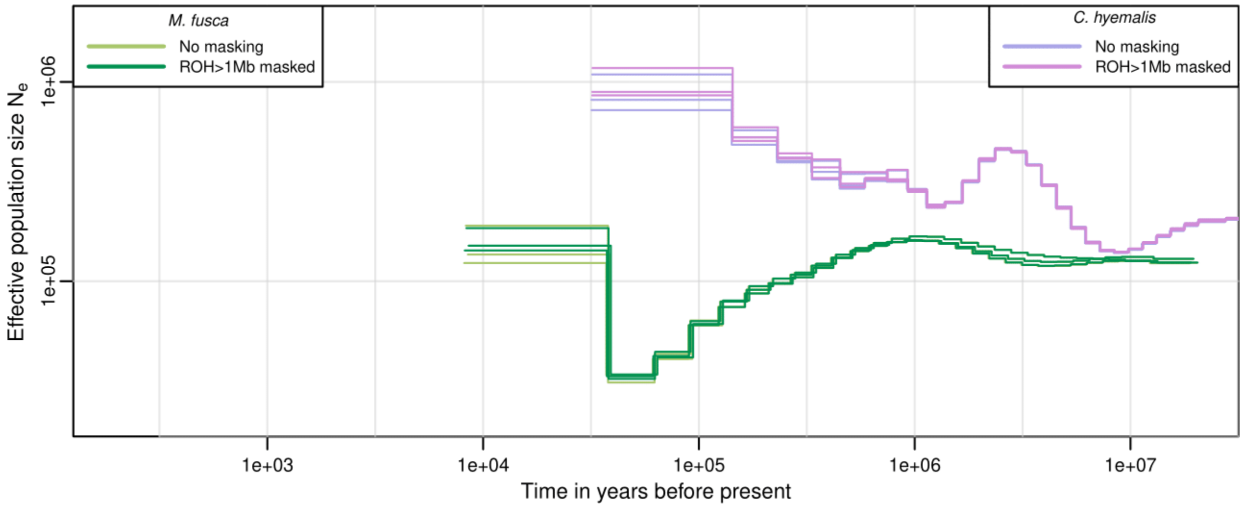

Figure S3. Effects of masking of ROH > 1Mb on PSMC trajectories for velvet scoter (*M. fusca,* green colors) and long-tailed duck (*C. hyemalis,* violet colors). Only the three most inbred individuals of each species are plotted. All analyses used a mutation rate of 4.6 x 10^{-9} and a generation time of 6.01 years *(C. hyemalis)* and 5.75 years *(M. fusca*).

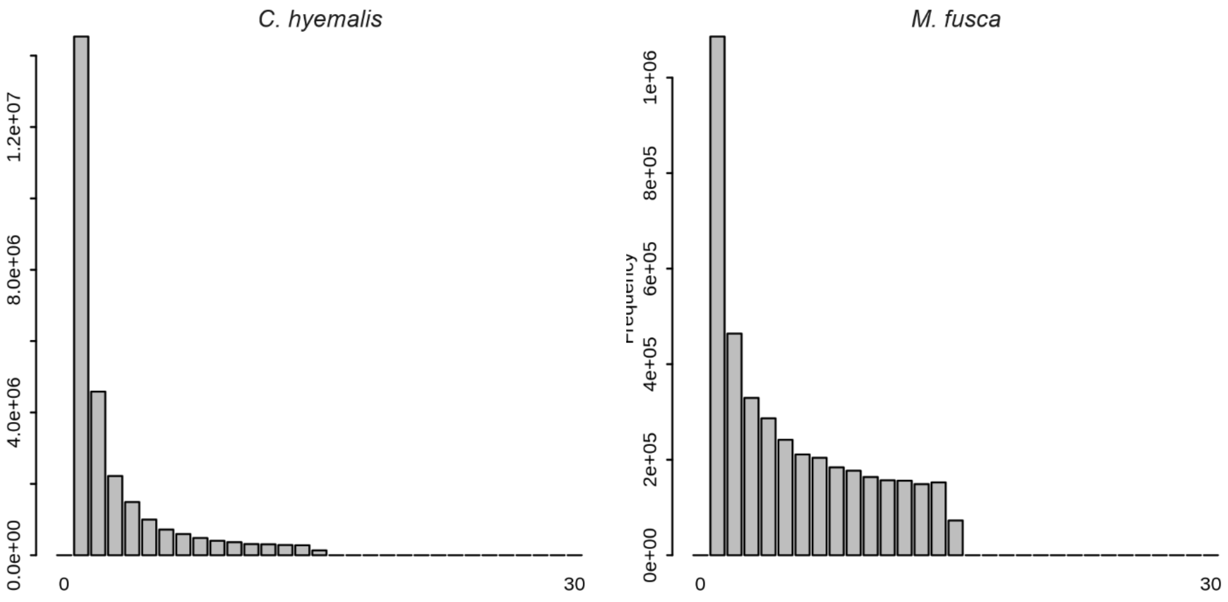

Figure S4. Visualization of folded site frequency spectra for *C. hyemalis* and *M. fusca*, respectively.

Figure S5. Runs of Homozygosity (ROH) detected along the 100 longest scaffolds* in *M. fusca*. The y-axis shows individuals, and the x-axis indicates chromosomal positions - note that the axis scale varies between plots due to differences in scaffold length.
*Only 49 scaffolds are shown as no ROH were detected in the remaining 51 scaffolds.

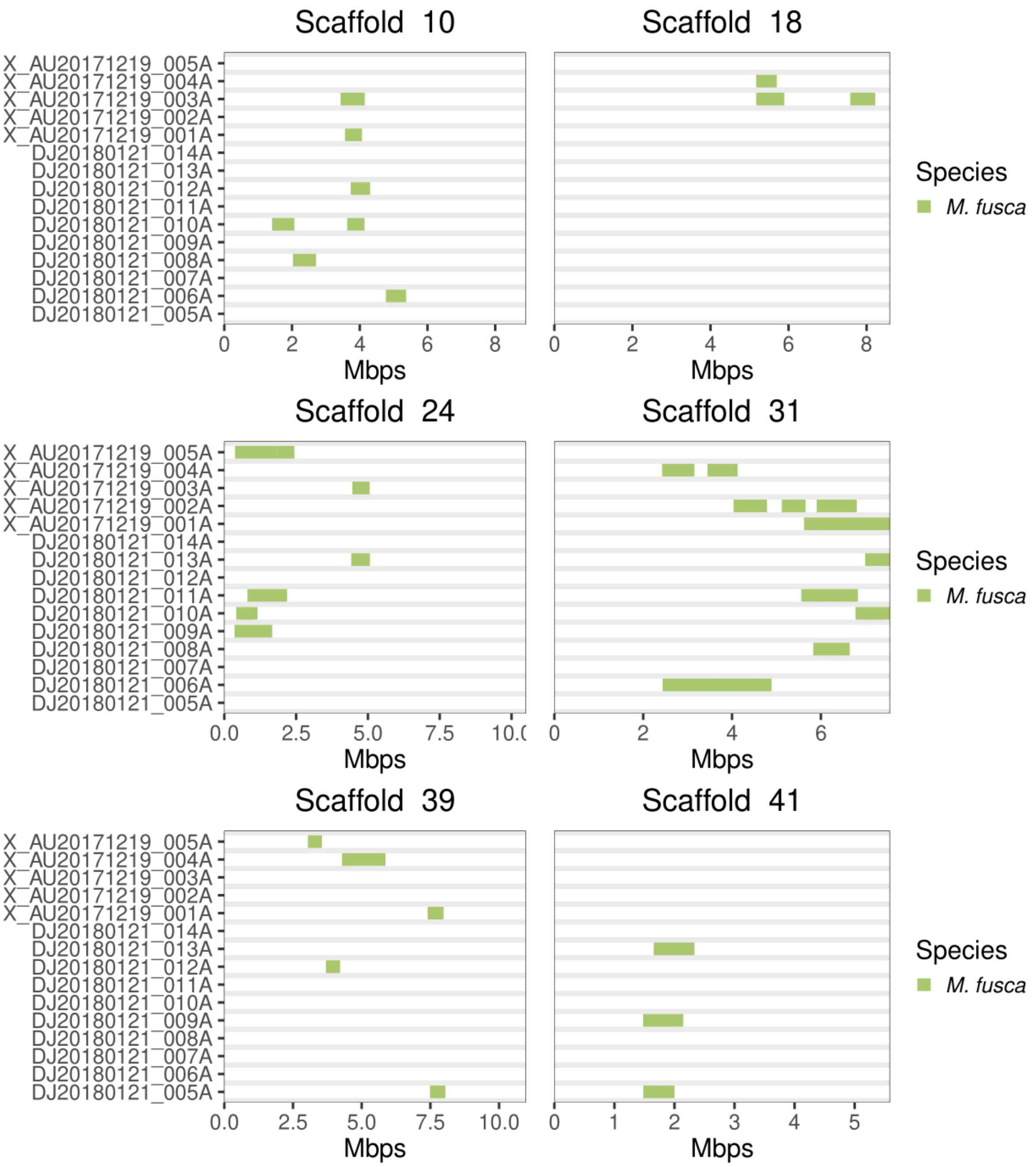

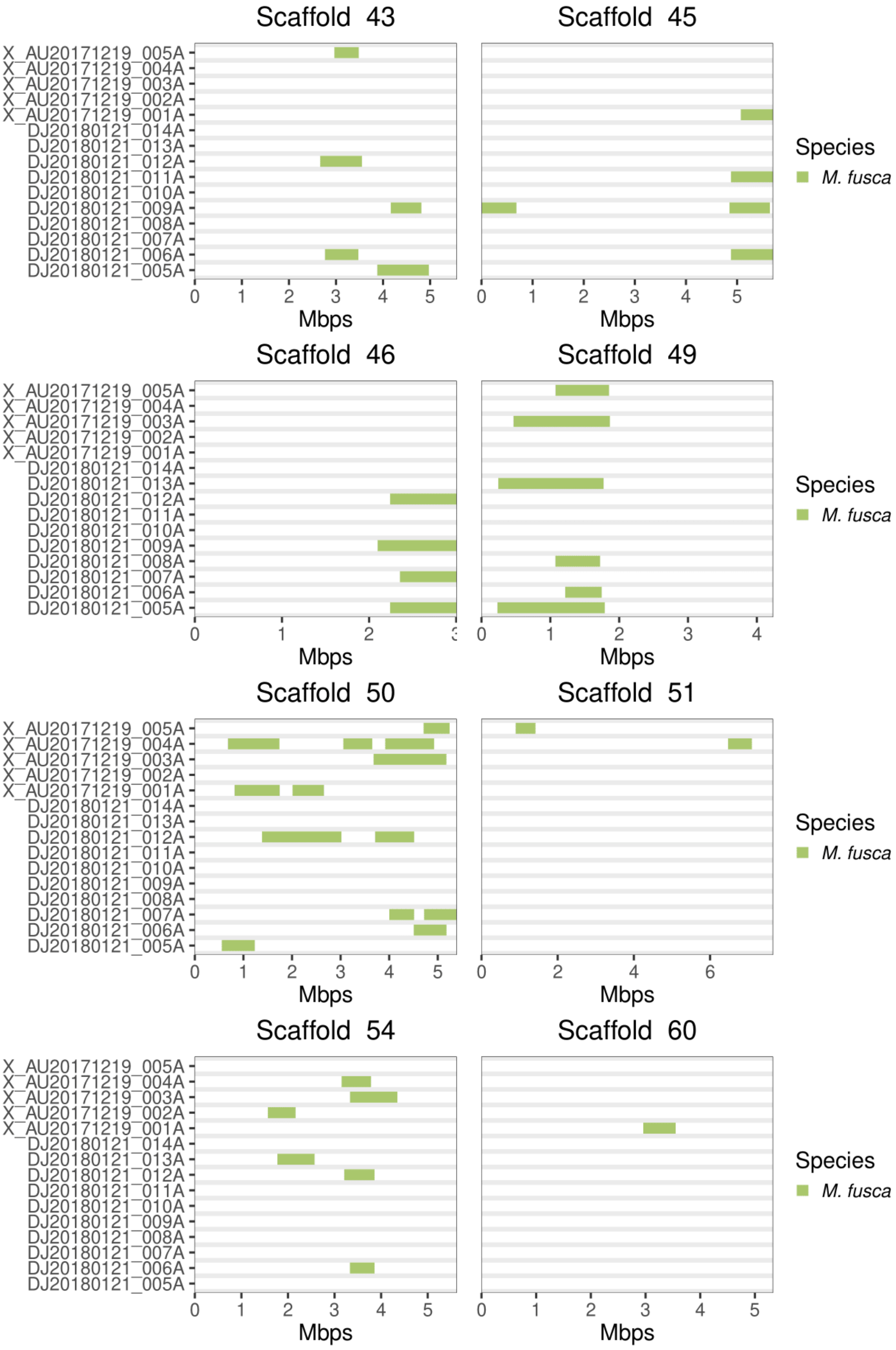

Scaffold 43
Scaffold 45
Scaffold 46
Scaffold 49
Scaffold 50
Scaffold 51
Scaffold 54
Scaffold 60
X_AU20171219_005A
X_AU20171219_004A
X_AU20171219_003A
X_AU20171219_002A
X_AU20171219_001A
DJ20180121_014A
DJ20180121_013A
DJ20180121_012A
DJ20180121_011A
DJ20180121_010A
DJ20180121_009A
DJ20180121_008A
DJ20180121_007A
DJ20180121_006A
DJ20180121_005A
Mbps
Species
M. fusca

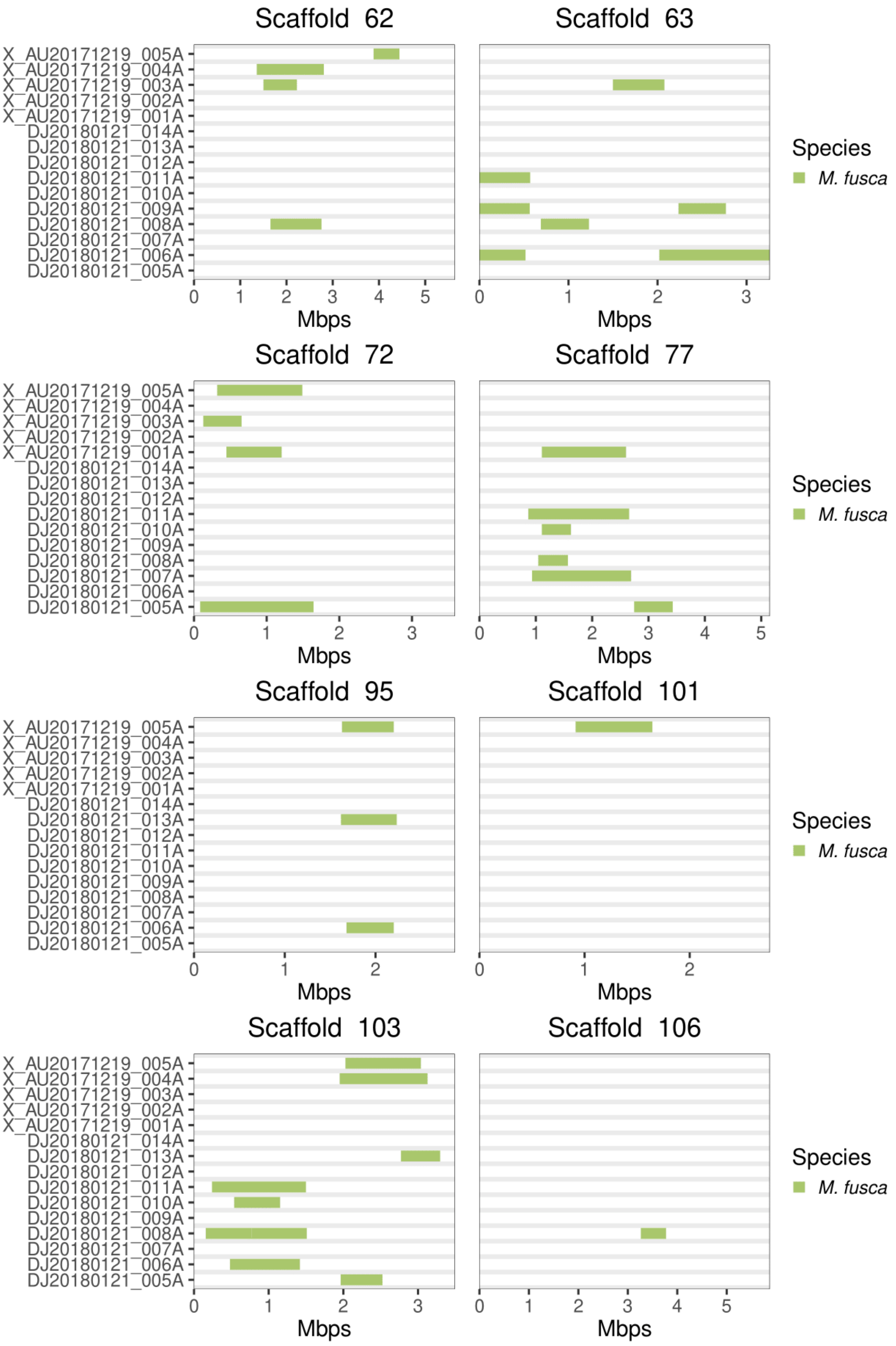

Scaffold 62
Scaffold 63
Scaffold 72
Scaffold 77
Scaffold 95
Scaffold 101
Scaffold 103
Scaffold 106
X_AU20171219_005A
X_AU20171219_004A
X_AU20171219_003A
X_AU20171219_002A
X_AU20171219_001A
DJ20180121_014A
DJ20180121_013A
DJ20180121_012A
DJ20180121_011A
DJ20180121_010A
DJ20180121_009A
DJ20180121_008A
DJ20180121_007A
DJ20180121_006A
DJ20180121_005A
Mbps
Species
M. fusca

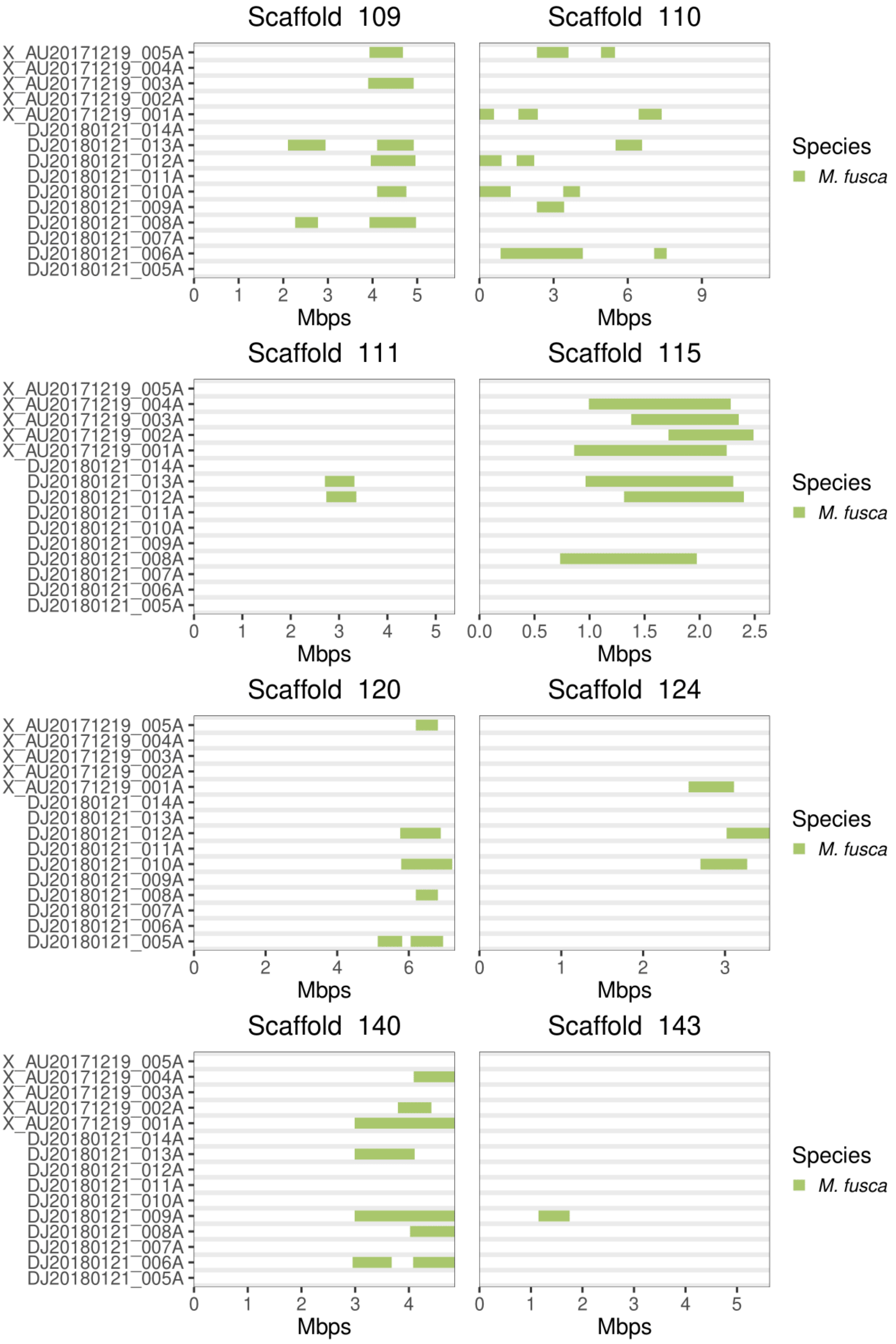
Scaffold 109
Scaffold 110
Scaffold 111
Scaffold 115
Scaffold 120
Scaffold 124
Scaffold 140
Scaffold 143
X_AU20171219_005A
X_AU20171219_004A
X_AU20171219_003A
X_AU20171219_002A
X_AU20171219_001A
DJ20180121_014A
DJ20180121_013A
DJ20180121_012A
DJ20180121_011A
DJ20180121_010A
DJ20180121_009A
DJ20180121_008A
DJ20180121_007A
DJ20180121_006A
DJ20180121_005A
Mbps
Species
M. fusca

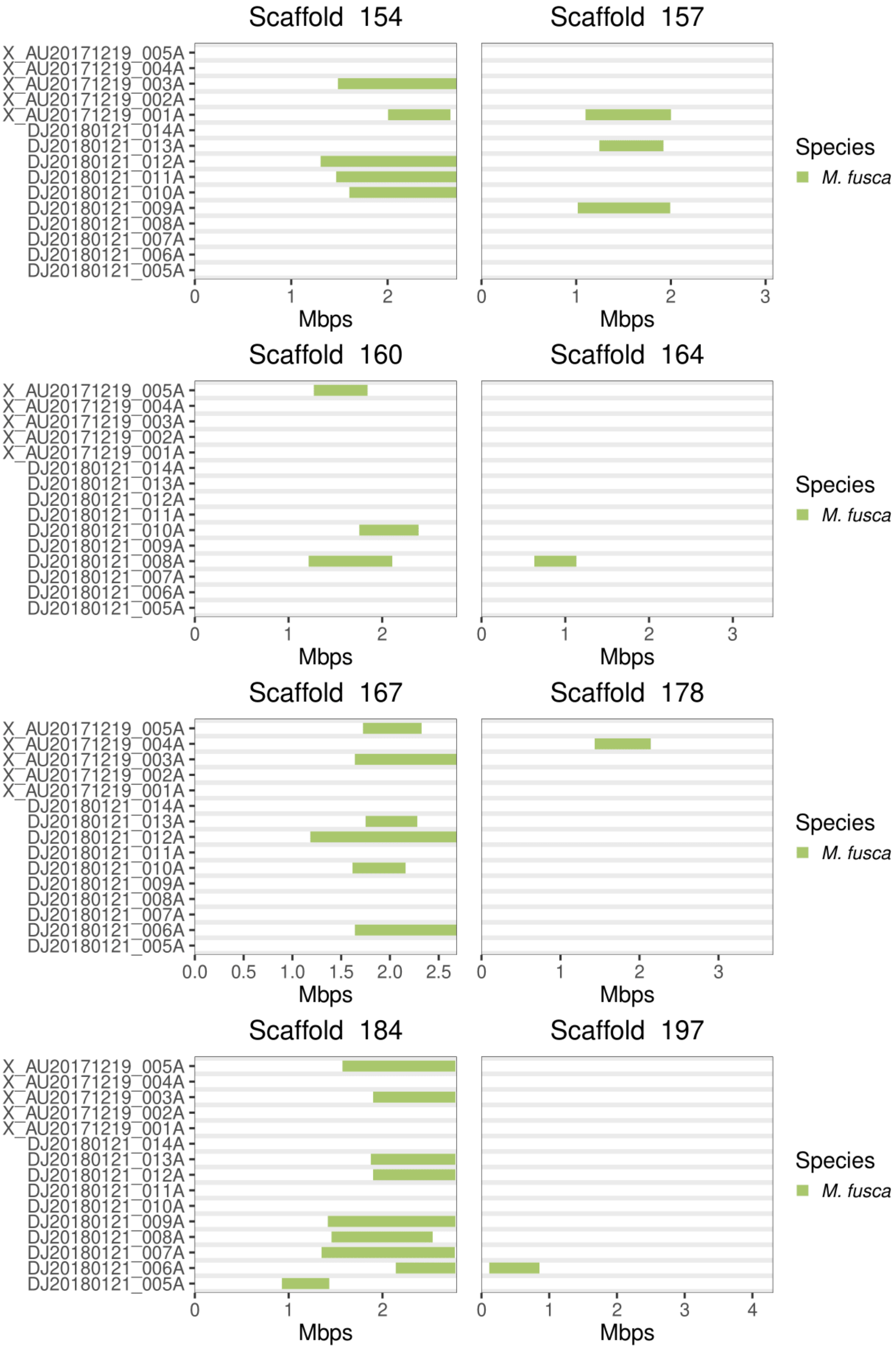

Scaffold 154
Scaffold 157
Scaffold 160
Scaffold 164
Scaffold 167
Scaffold 178
Scaffold 184
Scaffold 197
X_AU20171219_005A
X_AU20171219_004A
X_AU20171219_003A
X_AU20171219_002A
X_AU20171219_001A
DJ20180121_014A
DJ20180121_013A
DJ20180121_012A
DJ20180121_011A
DJ20180121_010A
DJ20180121_009A
DJ20180121_008A
DJ20180121_007A
DJ20180121_006A
DJ20180121_005A
Mbps
Species
M. fusca

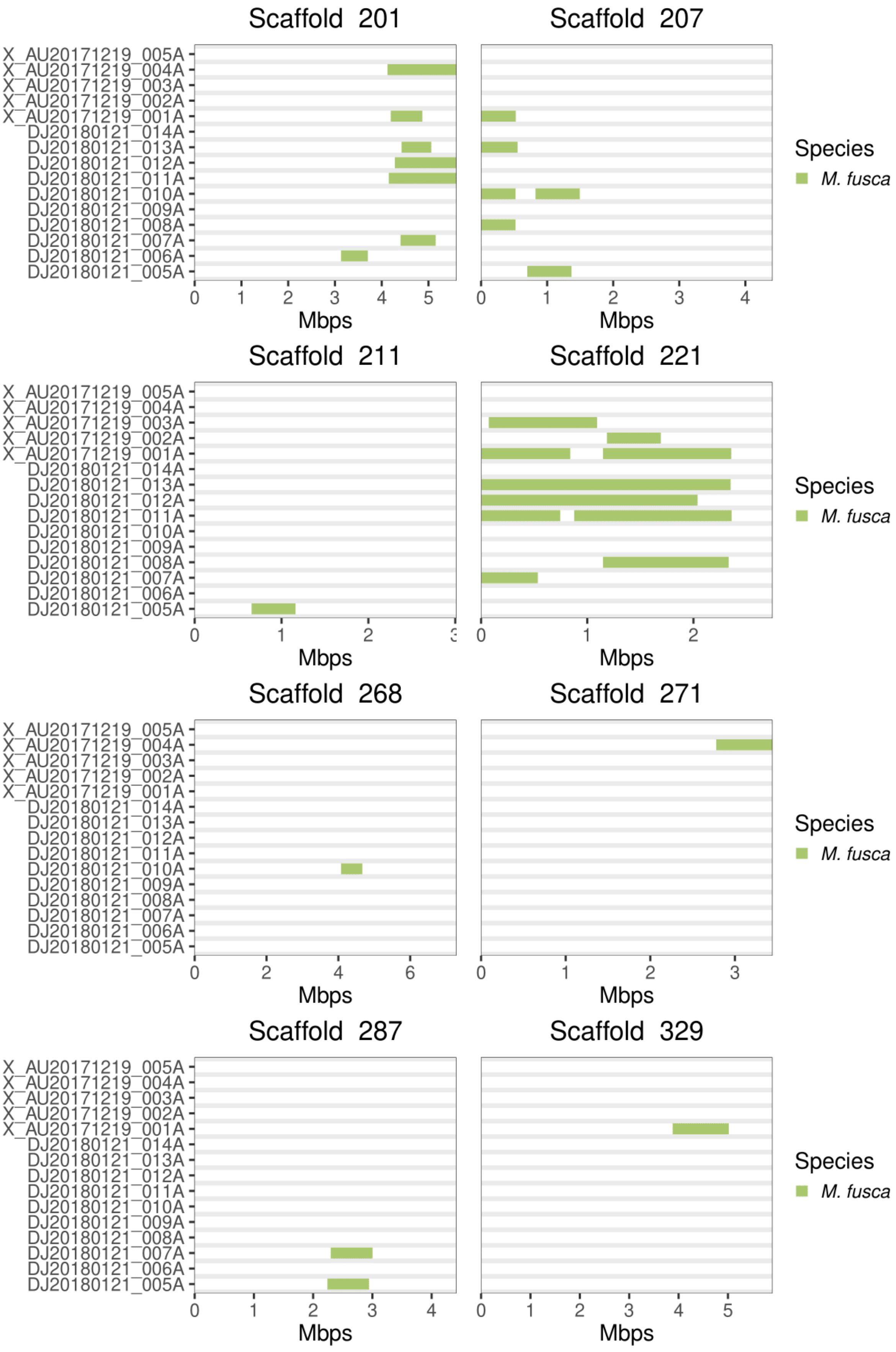

Scaffold 201
Scaffold 207
Scaffold 211
Scaffold 221
Scaffold 268
Scaffold 271
Scaffold 287
Scaffold 329
X_AU20171219_005A
X_AU20171219_004A
X_AU20171219_003A
X_AU20171219_002A
X_AU20171219_001A
DJ20180121_014A
DJ20180121_013A
DJ20180121_012A
DJ20180121_011A
DJ20180121_010A
DJ20180121_009A
DJ20180121_008A
DJ20180121_007A
DJ20180121_006A
DJ20180121_005A
Mbps
Species
M. fusca

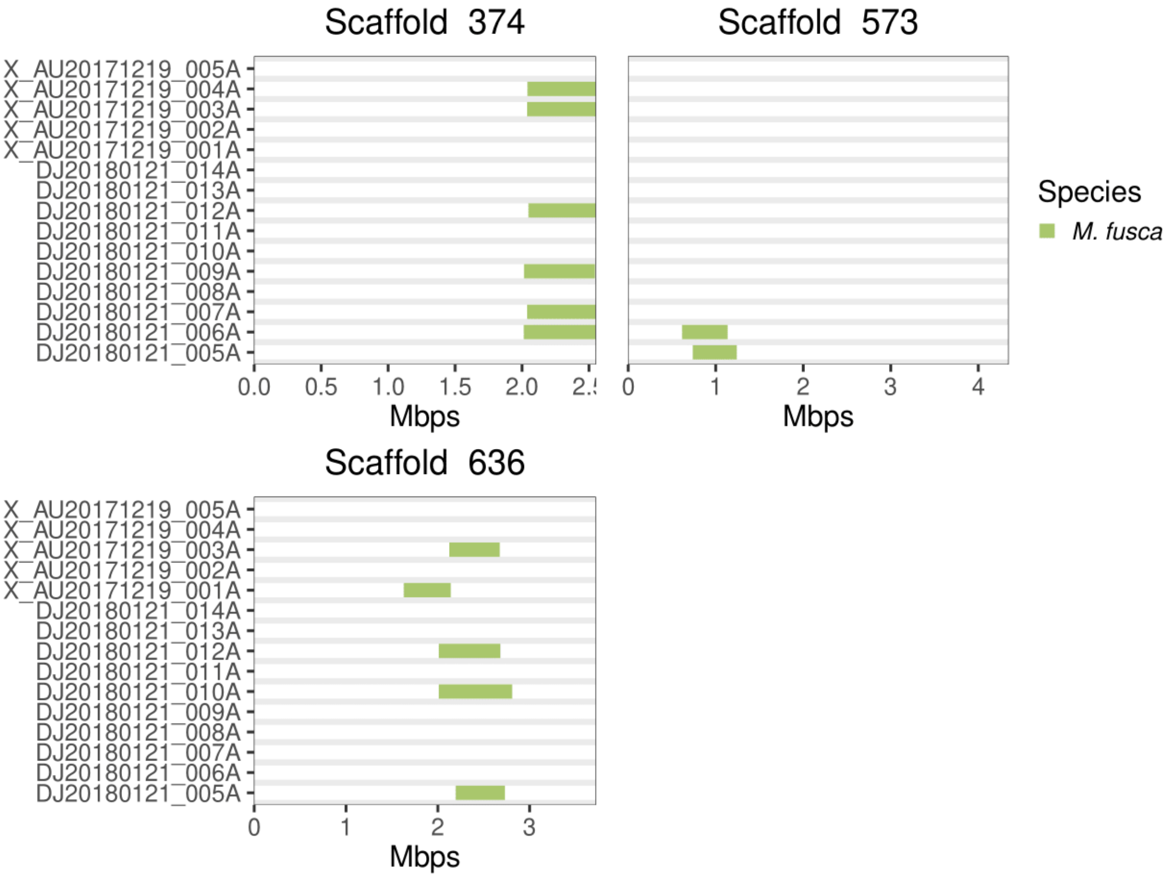
Scaffold 374
Scaffold 573
Scaffold 636
X_AU20171219_005A
X_AU20171219_004A
X_AU20171219_003A
X_AU20171219_002A
X_AU20171219_001A
DJ20180121_014A
DJ20180121_013A
DJ20180121_012A
DJ20180121_011A
DJ20180121_010A
DJ20180121_009A
DJ20180121_008A
DJ20180121_007A
DJ20180121_006A
DJ20180121_005A
0.0 0.5 1.0 1.5 2.0
0 1 2 3 4
0 1 2 3
Mbps
Species
M. fusca

Figure S6. Runs of Homozygosity (ROH) detected along the 100 longest scaffolds* in *C. hyemalis*. The y-axis shows individuals, and the x-axis indicates chromosomal positions - note that the axis scale varies between plots due to differences in scaffold length.
*Only 53 scaffolds are shown as no ROH were detected in the remaining 47 scaffolds.

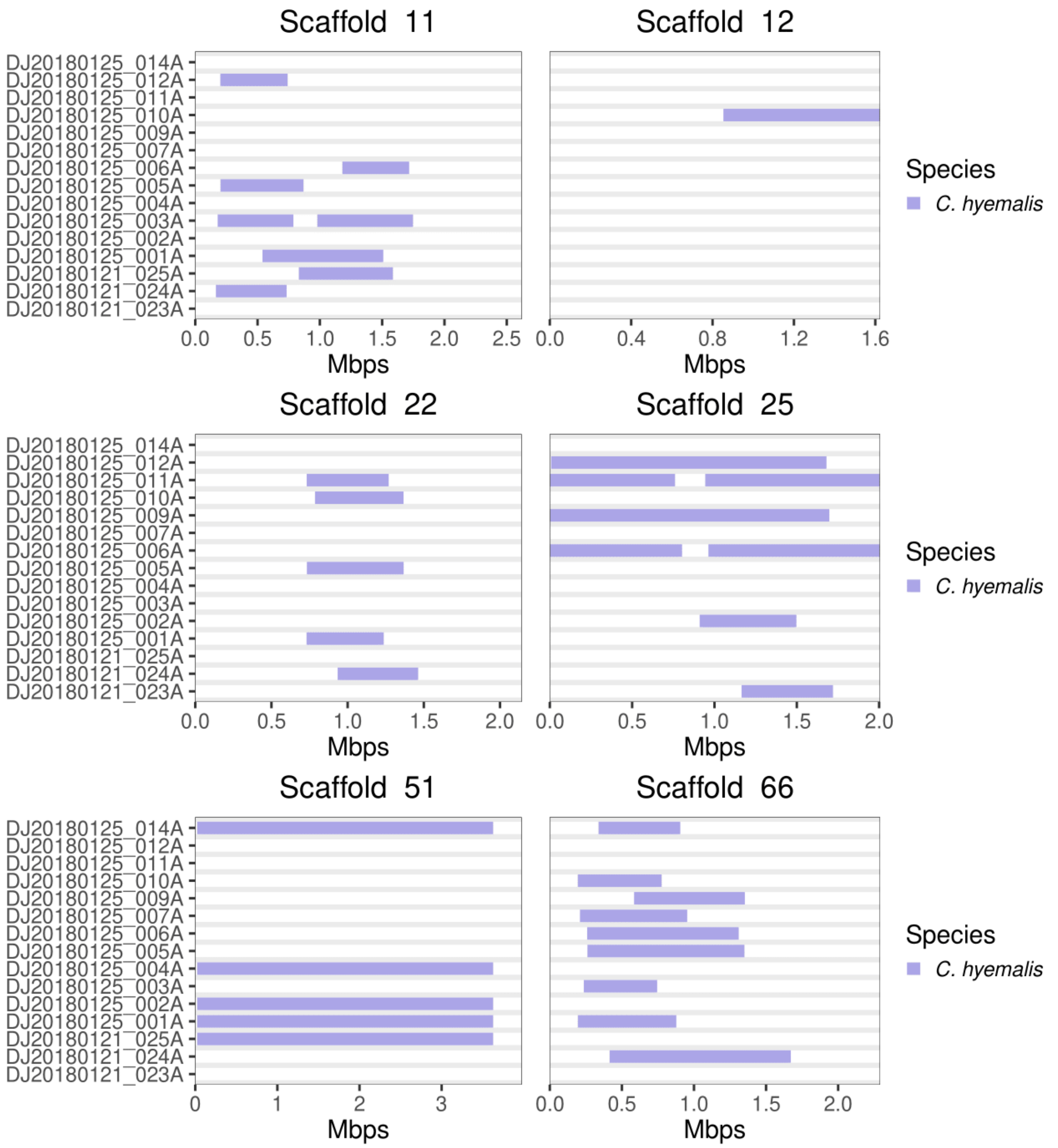

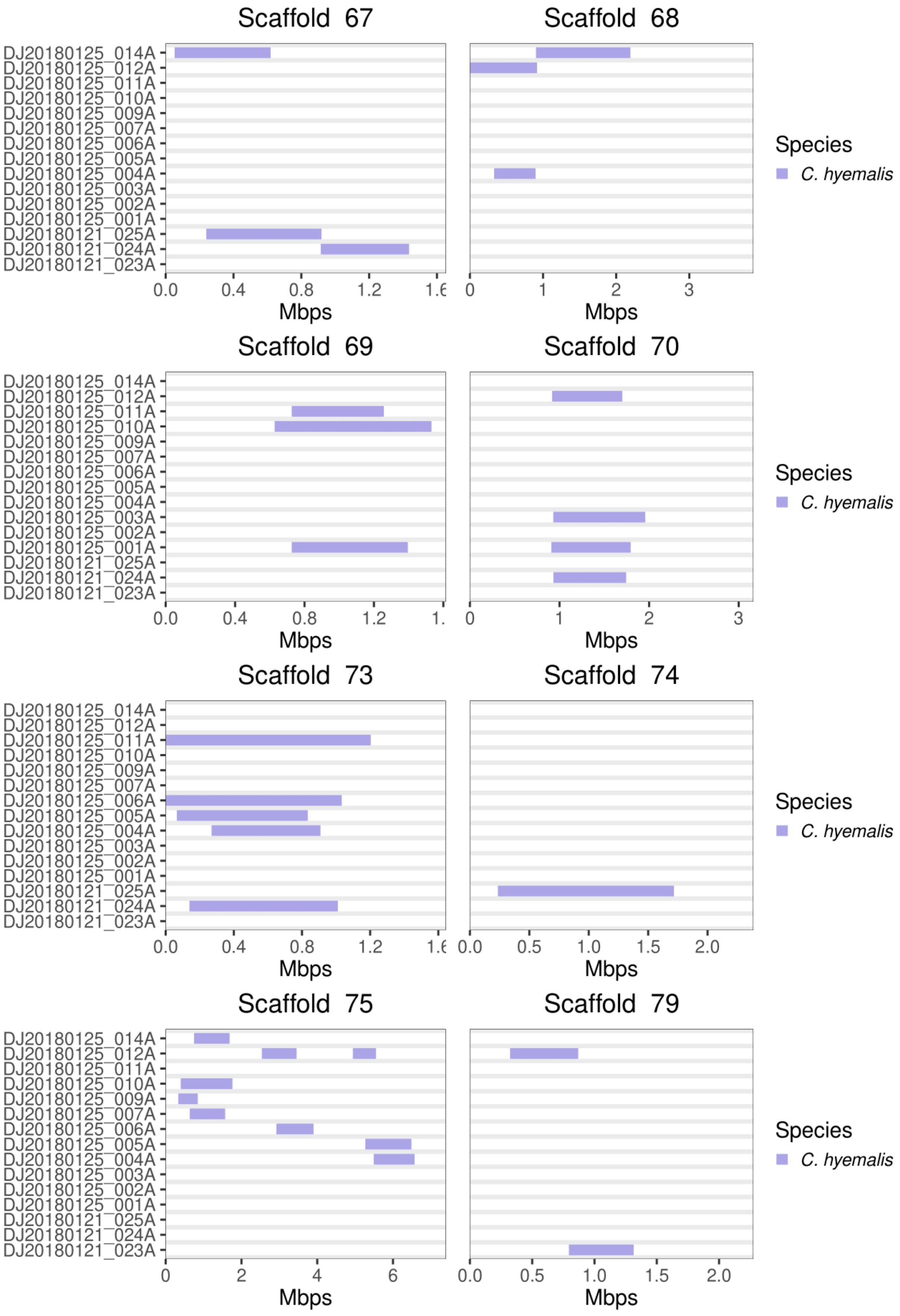
Scaffold 67
Scaffold 68
Scaffold 69
Scaffold 70
Scaffold 73
Scaffold 74
Scaffold 75
Scaffold 79
DJ20180125_014A
DJ20180125_012A
DJ20180125_011A
DJ20180125_010A
DJ20180125_009A
DJ20180125_007A
DJ20180125_006A
DJ20180125_005A
DJ20180125_004A
DJ20180125_003A
DJ20180125_002A
DJ20180125_001A
DJ20180121_025A
DJ20180121_024A
DJ20180121_023A
Mbps
0.0 0.4 0.8 1.2
0 1 2 3
0 2 4 6
0.0 0.5 1.0 1.5 2.0
Species
C. hyemalis

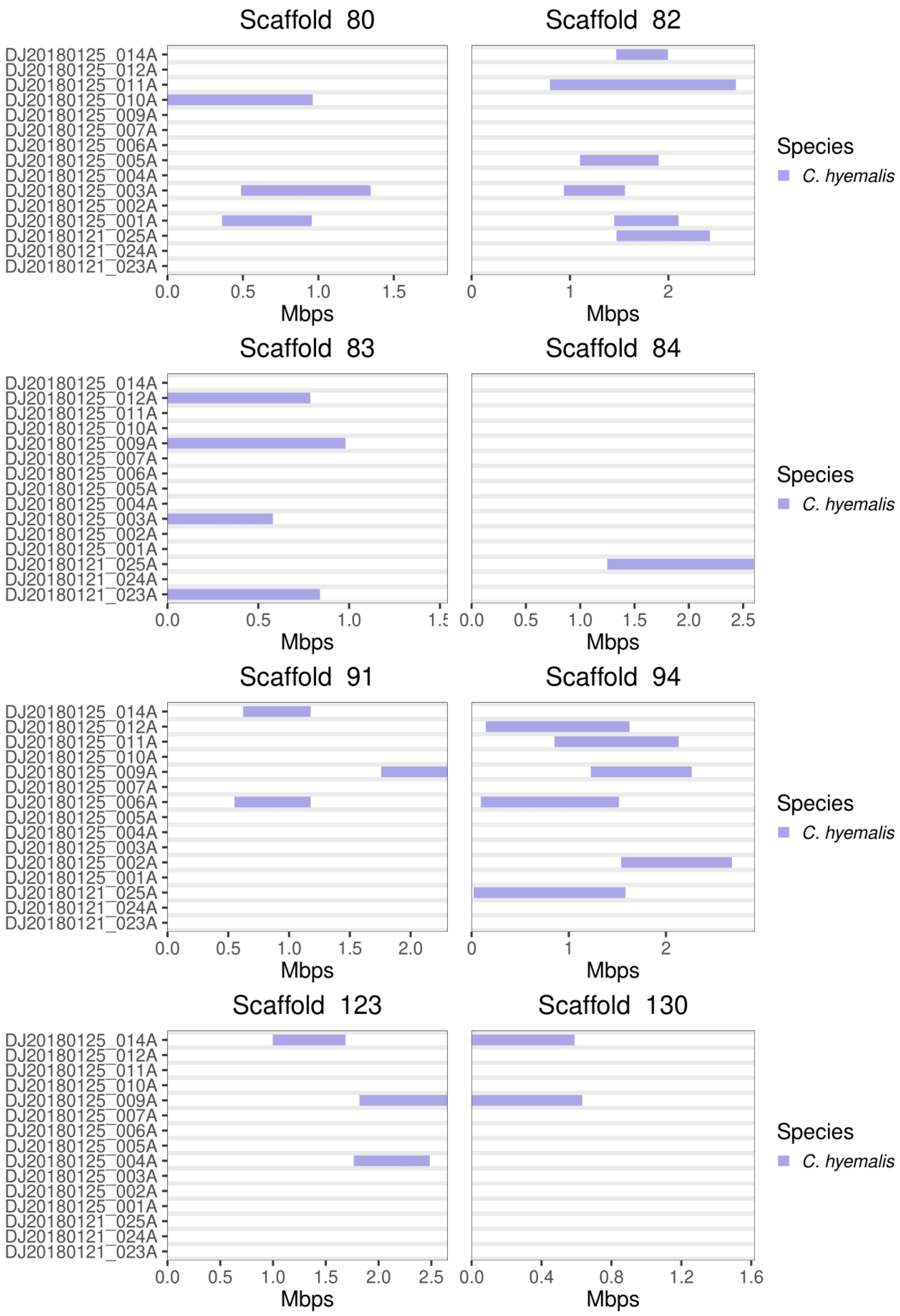

Scaffold 80
Scaffold 82
Scaffold 83
Scaffold 84
Scaffold 91
Scaffold 94
Scaffold 123
Scaffold 130
DJ20180125_014A
DJ20180125_012A
DJ20180125_011A
DJ20180125_010A
DJ20180125_009A
DJ20180125_007A
DJ20180125_006A
DJ20180125_005A
DJ20180125_004A
DJ20180125_003A
DJ20180125_002A
DJ20180125_001A
DJ20180121_025A
DJ20180121_024A
DJ20180121_023A
Mbps
Species
C. hyemalis

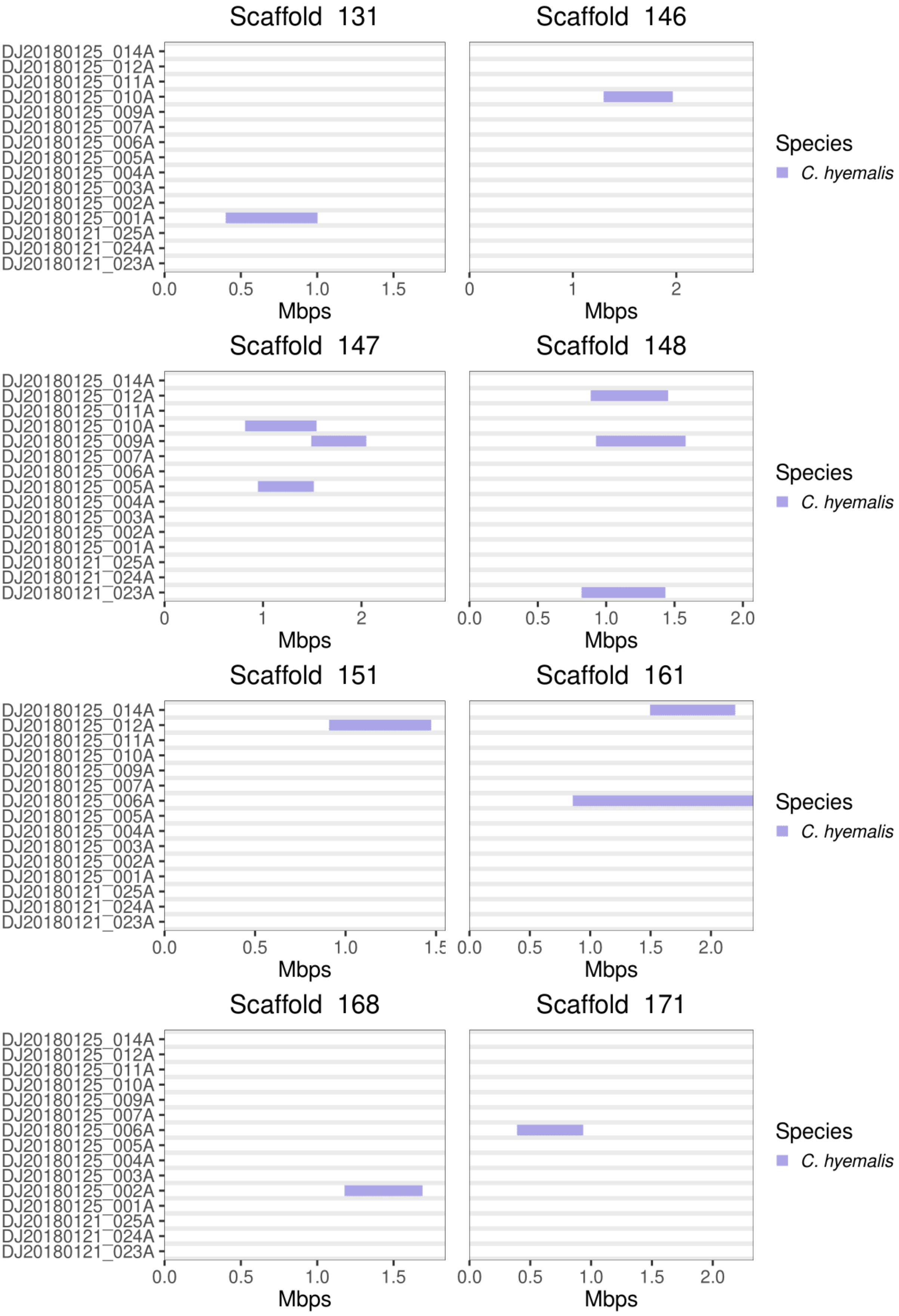

Scaffold 131
Scaffold 146
Scaffold 147
Scaffold 148
Scaffold 151
Scaffold 161
Scaffold 168
Scaffold 171
DJ20180125_014A
DJ20180125_012A
DJ20180125_011A
DJ20180125_010A
DJ20180125_009A
DJ20180125_007A
DJ20180125_006A
DJ20180125_005A
DJ20180125_004A
DJ20180125_003A
DJ20180125_002A
DJ20180125_001A
DJ20180121_025A
DJ20180121_024A
DJ20180121_023A
0.0 0.5 1.0 1.5
0 1 2
0.0 0.5 1.0 1.5 2.0
Mbps
Species
C. hyemalis

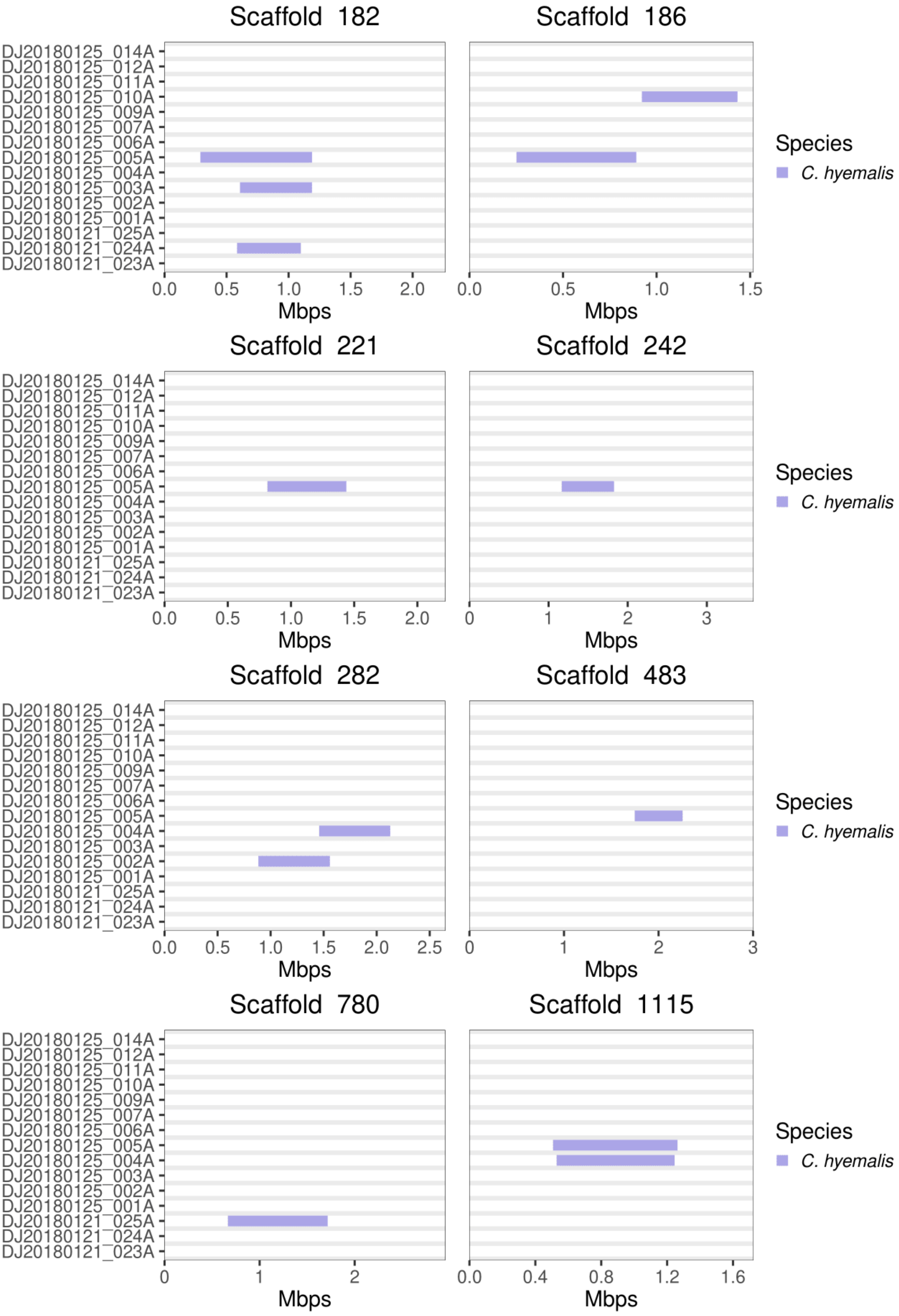
Scaffold 182
Scaffold 186
Scaffold 221
Scaffold 242
Scaffold 282
Scaffold 483
Scaffold 780
Scaffold 1115
DJ20180125_014A
DJ20180125_012A
DJ20180125_011A
DJ20180125_010A
DJ20180125_009A
DJ20180125_007A
DJ20180125_006A
DJ20180125_005A
DJ20180125_004A
DJ20180125_003A
DJ20180125_002A
DJ20180125_001A
DJ20180121_025A
DJ20180121_024A
DJ20180121_023A
Mbps
Species
C. hyemalis

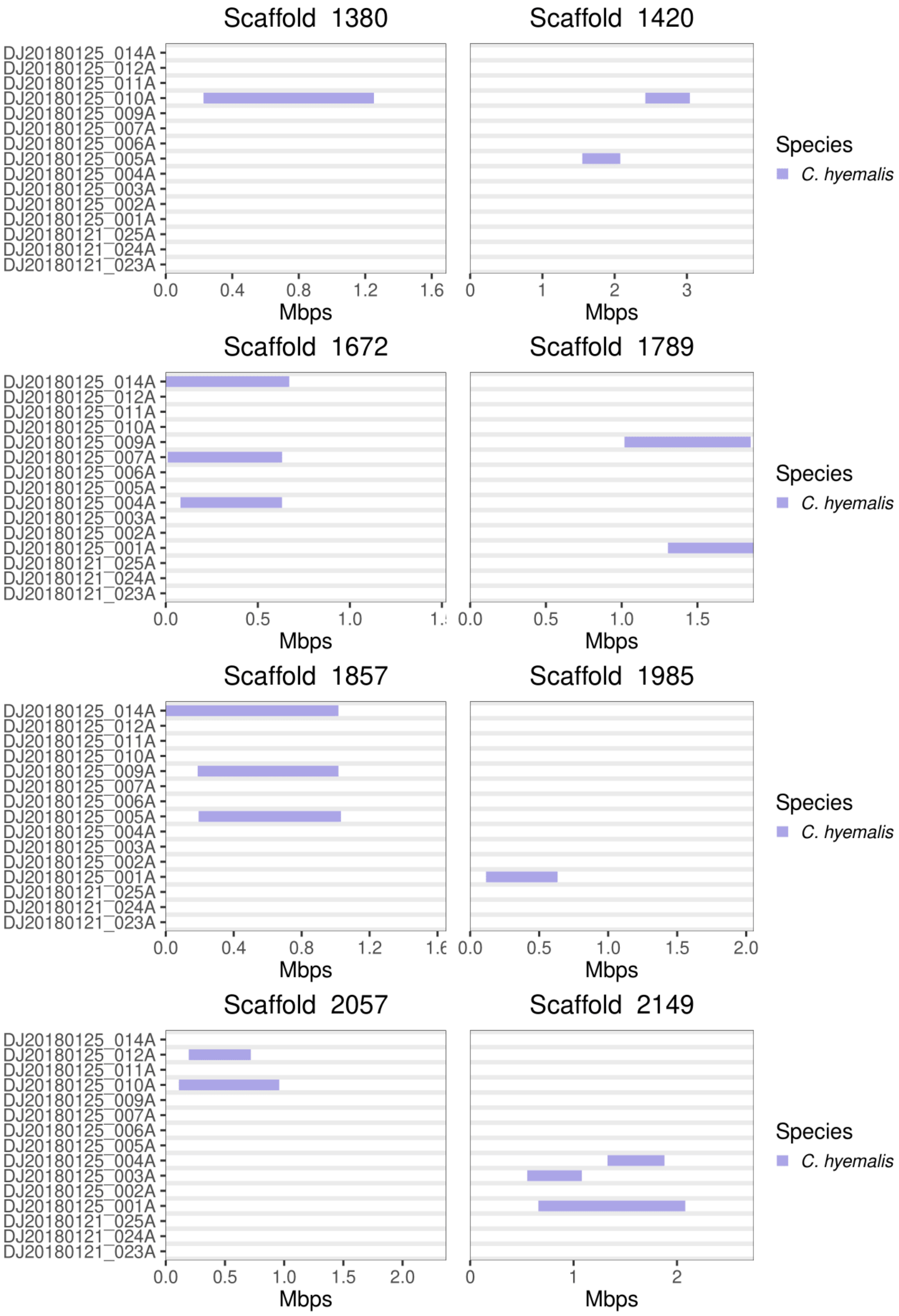
Scaffold 1380
Scaffold 1420
Scaffold 1672
Scaffold 1789
Scaffold 1857
Scaffold 1985
Scaffold 2057
Scaffold 2149
DJ20180125_014A
DJ20180125_012A
DJ20180125_011A
DJ20180125_010A
DJ20180125_009A
DJ20180125_007A
DJ20180125_006A
DJ20180125_005A
DJ20180125_004A
DJ20180125_003A
DJ20180125_002A
DJ20180125_001A
DJ20180121_025A
DJ20180121_024A
DJ20180121_023A
0.0 0.4 0.8 1.2 1.6
0 1 2 3
0.0 0.5 1.0
0.0 0.5 1.0 1.5
0.0 0.4 0.8 1.2
0.0 0.5 1.0 1.5 2.0
0.0 0.5 1.0 1.5 2.0
0 1 2
Mbps
Species
C. hyemalis

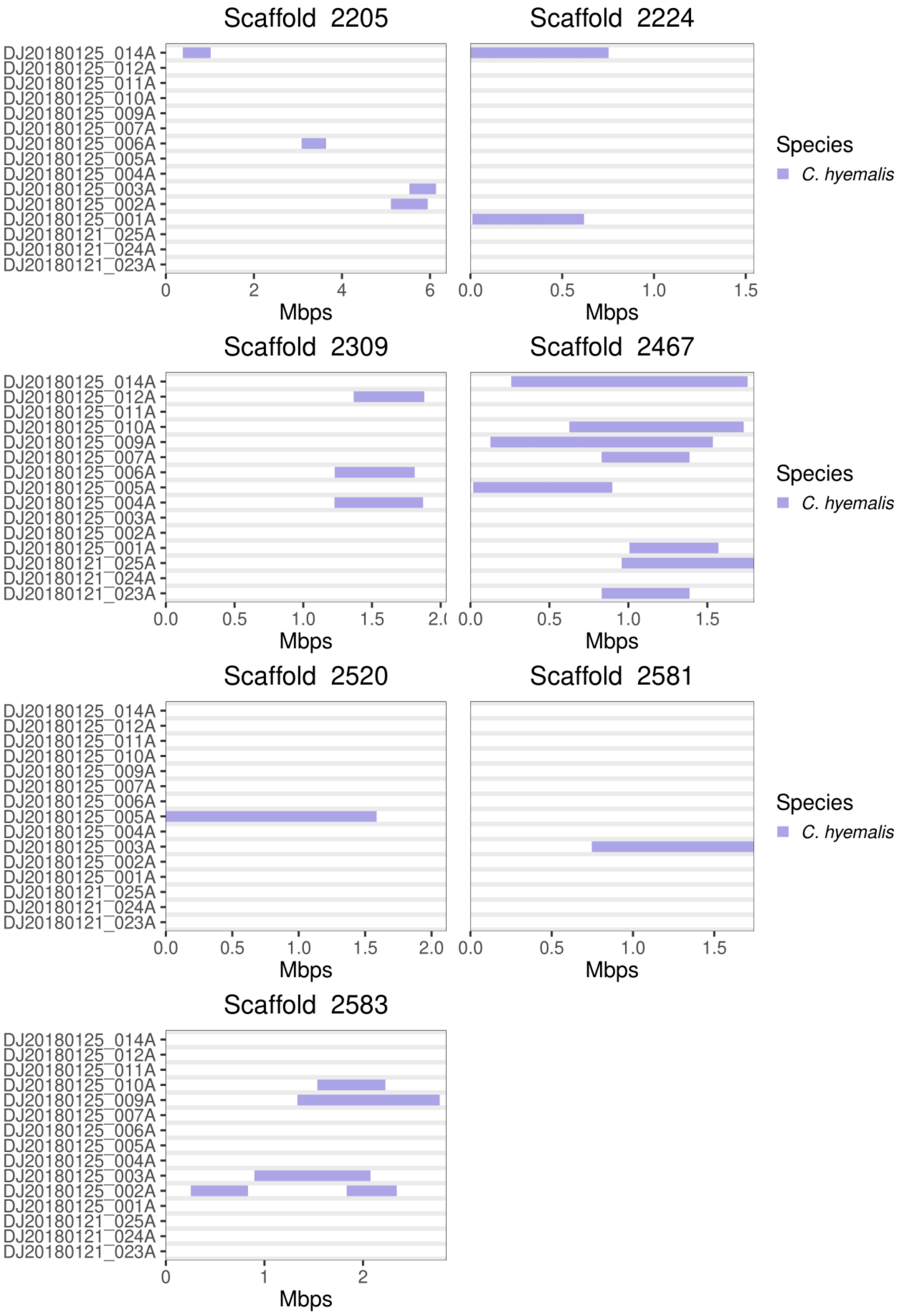

Scaffold 2205
Scaffold 2224
DJ20180125_014A
DJ20180125_012A
DJ20180125_011A
DJ20180125_010A
DJ20180125_009A
DJ20180125_007A
DJ20180125_006A
DJ20180125_005A
DJ20180125_004A
DJ20180125_003A
DJ20180125_002A
DJ20180125_001A
DJ20180121_025A
DJ20180121_024A
DJ20180121_023A
0
2
4
6
Mbps
0.0
0.5
1.0
1.5
Mbps
Species
C. hyemalis
Scaffold 2309
Scaffold 2467
DJ20180125_014A
DJ20180125_012A
DJ20180125_011A
DJ20180125_010A
DJ20180125_009A
DJ20180125_007A
DJ20180125_006A
DJ20180125_005A
DJ20180125_004A
DJ20180125_003A
DJ20180125_002A
DJ20180125_001A
DJ20180121_025A
DJ20180121_024A
DJ20180121_023A
0.0
0.5
1.0
1.5
2.(
Mbps
0.0
0.5
1.0
1.5
Mbps
Species
C. hyemalis
Scaffold 2520
Scaffold 2581
DJ20180125_014A
DJ20180125_012A
DJ20180125_011A
DJ20180125_010A
DJ20180125_009A
DJ20180125_007A
DJ20180125_006A
DJ20180125_005A
DJ20180125_004A
DJ20180125_003A
DJ20180125_002A
DJ20180125_001A
DJ20180121_025A
DJ20180121_024A
DJ20180121_023A
0.0
0.5
1.0
1.5
2.0
Mbps
0.0
0.5
1.0
1.5
Mbps
Species
C. hyemalis
Scaffold 2583
DJ20180125_014A
DJ20180125_012A
DJ20180125_011A
DJ20180125_010A
DJ20180125_009A
DJ20180125_007A
DJ20180125_006A
DJ20180125_005A
DJ20180125_004A
DJ20180125_003A
DJ20180125_002A
DJ20180125_001A
DJ20180121_025A
DJ20180121_024A
DJ20180121_023A
0
1
2
Mbps
